# Supplementary material for: Microtubule Nucleation Properties of Single Human γTuRCs Explained by Their Cryo-EM Structure
Source: Dev Cell. 2020 Jun 8;53(5):603–617.e8. doi: 10.1016/j.devcel.2020.04.019 (PMC7280788; doi:10.1016/j.devcel.2020.04.019)
Supplement: Document S1. Figures S1–S6 [file mmc1.pdf]

Developmental Cell, Volume *53*

Supplemental Information

Microtubule Nucleation Properties of Single Human γTuRCs Explained by Their Cryo-EM Structure

Tanja Consolati, Julia Locke, Johanna Roostalu, Zhuo Angel Chen, Julian Gannon, Jayant Asthana, Wei Ming Lim, Fabrizio Martino, Milos A. Cvetkovic, Juri Rappsilber, Alessandro Costa, and Thomas Surrey

Figure S1

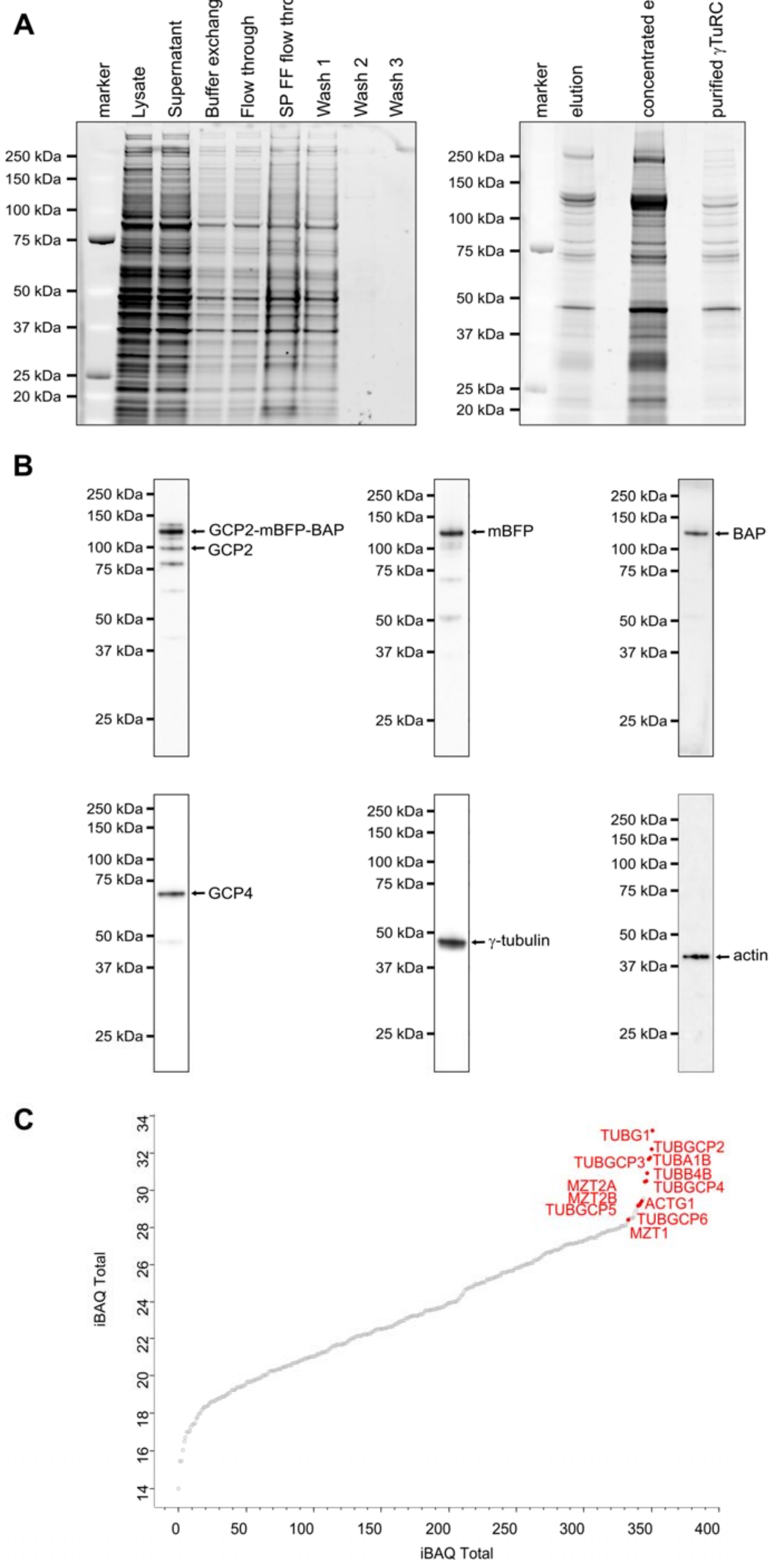
A
marker
Lysate
Supernatant
Buffer exchange
Flow through
SP FF flow through
Wash 1
Wash 2
Wash 3
250 kDa
150 kDa
100 kDa
75 kDa
50 kDa
37 kDa
25 kDa
20 kDa
marker
elution
concentrated elution
purified γTuRC
B
GCP2-mBFP-BAP
GCP2
mBFP
BAP
GCP4
γ-tubulin
actin
C
TUBG1
TUBGCP2
TUBGCP3
TUBA1B
TUBB4B
TUBGCP4
MZT2A
MZT2B
TUBGCP5
ACTG1
TUBGCP6
MZT1
iBAQ Total
iBAQ Total

Figure S1. Purification of tagged human γTuRC (Related to Fig. 1). (A) Sypro-Ruby stained SDS-PAGE showing the purification of γTuRC-mBFP-BAP purification from human cells. **(B)** Uncropped western blots of purified γTuRC using antibodies against GCP2, mBFP, GCP4, γ-tubulin and actin. Biotinylation of the biotin acceptor peptide (BAP) was assessed by immunoblotting using horse radish peroxidase (HRP)-coupled streptavidin. **(C)** Scatter plot representing the protein density by iBAQ intensity. Rank vs log10 ibaq value ordered from lowest to highest estimated relative abundance. TUBG1 was the most abundant protein in the prep.

Figure S2

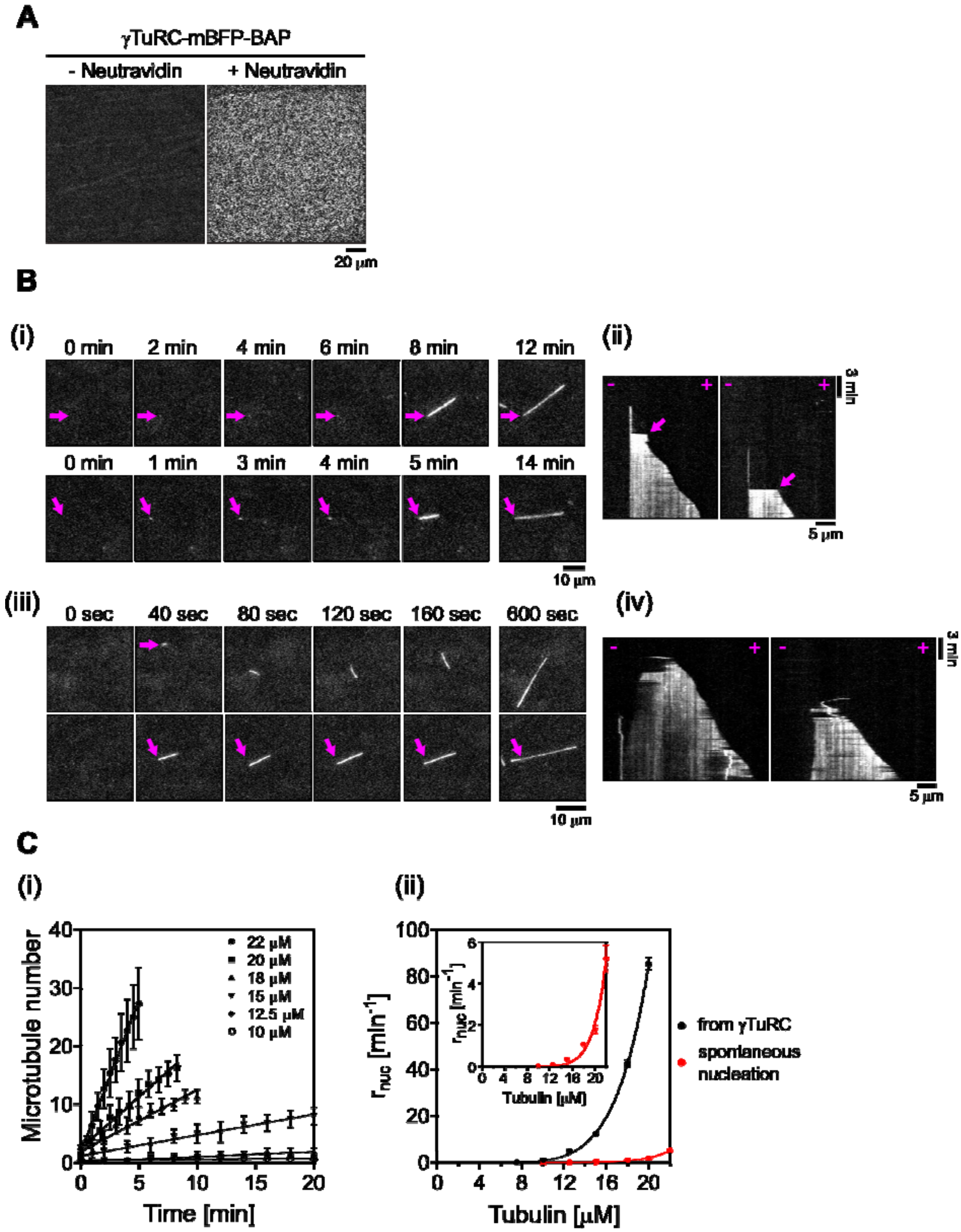
A
γTuRC-mBFP-BAP
- Neutravidin
+ Neutravidin
20 µm
B
(i)
0 min
2 min
4 min
6 min
8 min
12 min
0 min
1 min
3 min
4 min
5 min
14 min
10 µm
(ii)
3 min
5 µm
(iii)
0 sec
40 sec
80 sec
120 sec
160 sec
600 sec
10 µm
(iv)
3 min
5 µm
C
(i)
Microtubule number
Time [min]
22 µM
20 µM
18 µM
15 µM
12.5 µM
10 µM
(ii)
r_nuc [min^-1]
Tubulin [µM]
from γTuRC
spontaneous nucleation

Figure S2. Characterization of TIRF-microscopy based microtubule nucleation assay in the presence and absence of γTuRC (Related to Figs. 1, 2 and 3). (A) Specificity of γTuRC immobilization on functionalized glass surfaces. Biotinylated fluorescent γTuRC (373 pM used for immobilization) was bound to functionalized glass surfaces pre-incubated with NeutrAvidin or in the absence of NeutrAvidin as indicated. Representative TIRFM images of γTuRC-mBFP fluorescence are shown, indicating that γTuRC surface immobilization is specific. No tubulin was added. Field of view was always 164 µm x 164 µm. Fluorescence intensities are directly comparable. Scale bars as indicated. **(B)** Examples of 'falling' γTuRC-nucleated microtubules and 'landing' spontaneously nucleated microtubules in solution. **(i)** Representative time series of two γTuRC-nucleated microtubules that first grow out of the TIRF field and then 'fall' onto the surface (15 µM CF640R-tubulin, 93 pM γTuRC, 33°C). 'Falling' microtubules only grow from one end while the other end is stably anchored to the surface indicating that these microtubules are initiated by surface immobilized γTuRC. Purple arrows indicate the stably anchored microtubule ends. **(ii)** Corresponding TIRFM kymographs. Purple arrow marks 'falling' event. **(iii)** Representative time series of two spontaneously nucleated microtubules, 'landing' from solution (15 µM CF640R-tubulin, 47 pM γTuRC, 33°C). Spontaneously nucleated microtubules are distinguishable from γTuRC-nucleated microtubules because they are not anchored to the surface (top row, purple arrow mark first appearance of spontaneously nucleated microtubule) and grow with both ends (bottom row, purple arrows mark slow growing minus-end). **(iv)** Corresponding TIRFM kymographs. Scale bars as indicated. t=0 is 2 min after placing the sample at 33°C. **(C)** Dependence of spontaneous microtubule nucleation in the absence of γTuRC on tubulin concentration. **(i)** Microtubule nucleation at 33°C in the absence of γTuRC at varying CF640R-tubulin concentrations (10 µM, 12.5 µM, 15 µM, 18 µM, 20 µM, 22 µM). Plot is showing the linear increase in microtubule number over time. No microtubules were nucleated below 10 µM tubulin. Lines represent a linear regression. **(ii)** Plot showing the mean microtubule nucleation rate at different tubulin concentrations in the presence of γTuRC (see Fig. 3d (iv), black) and the mean rate of spontaneous microtubule nucleation in the absence of γTuRC at different tubulin concentrations (red, and inset). Data for plots were pooled from at least three independent experiments. The plots were fit using a power law function. Nucleation rates (r_{nuc}) were taken from the slope of the linear regression of the increase of microtubule number over time (see Fig. S6a and Fig. 3d (i)). All error bars are s.e.m. For symbols without visible error bars, error bars are smaller than the symbol size. t=0 is 2 min after placing the sample at 33 °C.

Figure S3

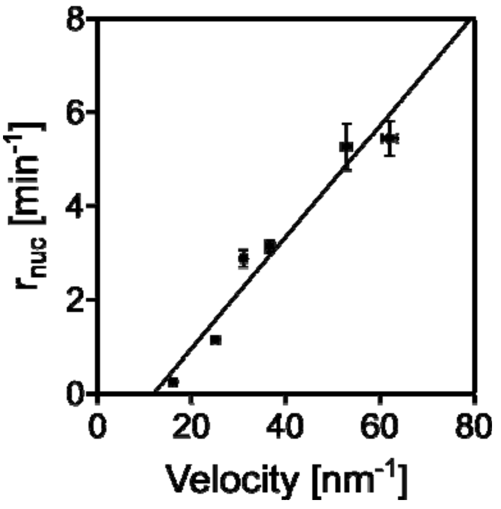

Figure S3. Linear relationship between γTuRC-mediated microtubule nucleation and chTOG-accelerated microtubule plus-end growth (Related to Fig. 4). Plot of the mean nucleation rate (r_{nuc}) against the mean microtubule plus-end growth speed at different chTOG-mGFP concentrations (6 nM, 13 nM, 25 nM, 50 nM, 100 nM). Assays were performed as described in Fig.4 a-d. Nucleation rates (r_{nuc}) were taken from Fig. 4b and microtubule growth speeds from Fig. 4d. Line represents the linear regression. Error bars are s.e.m. For symbols without visible error bars, error bars are smaller than the symbol size.

Figure S4

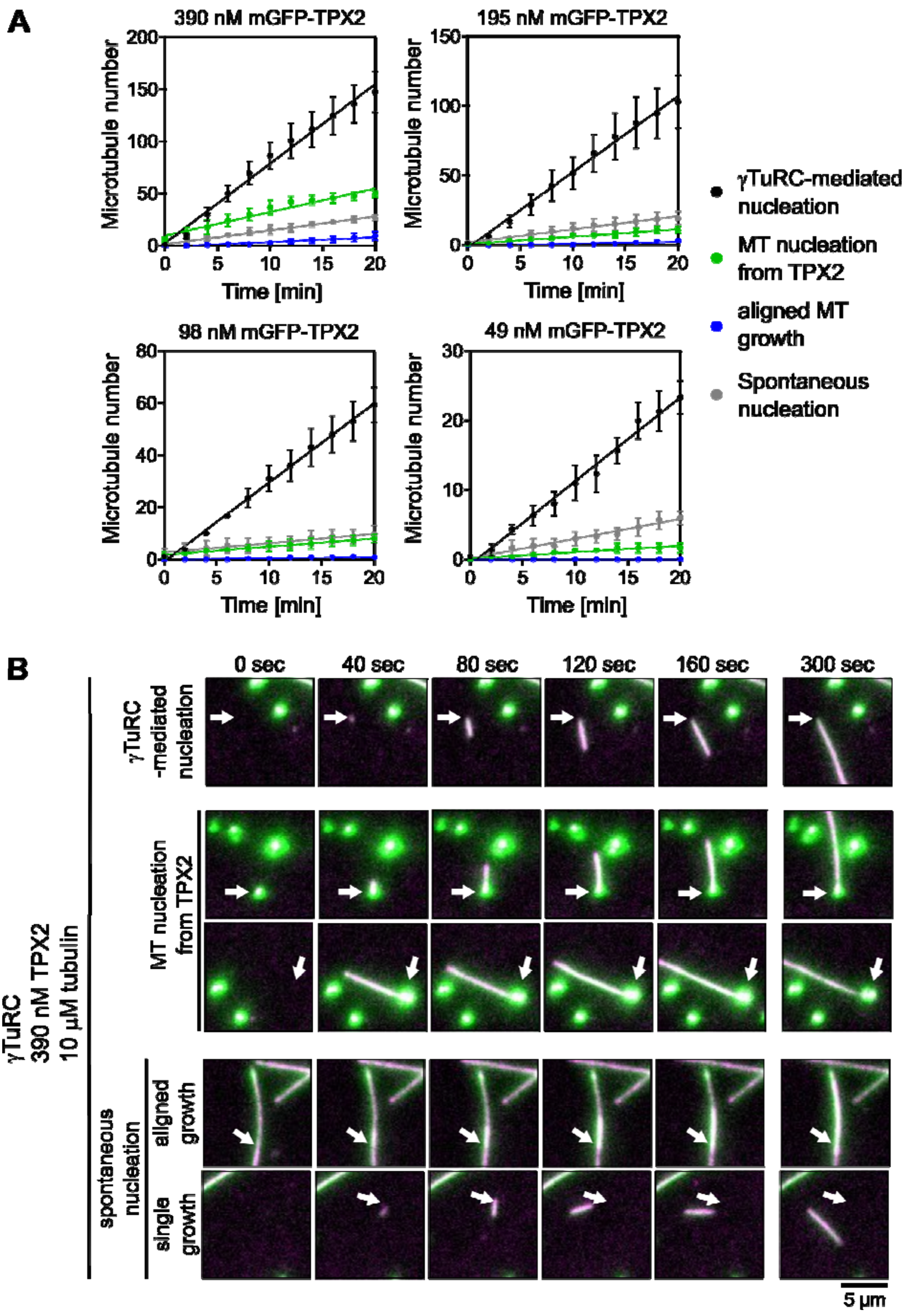
A
390 nM mGFP-TPX2
195 nM mGFP-TPX2
98 nM mGFP-TPX2
49 nM mGFP-TPX2
Microtubule number
Time [min]
γTuRC-mediated nucleation
MT nucleation from TPX2
aligned MT growth
Spontaneous nucleation
B
0 sec
40 sec
80 sec
120 sec
160 sec
300 sec
γTuRC -mediated nucleation
MT nucleation from TPX2
spontaneous nucleation
aligned growth
single growth
γTuRC
390 nM TPX2
10 μM tubulin
5 μm

Figure S4. Microtubules nucleated in assays performed in the presence of mGFP-TPX2 (Related to Fig. 4). (A) Plots showing linearly increasing nucleated microtubule numbers over time at different mGFP-TPX2 concentrations (49 nM, 98 nM, 195 nM, 390 nM). Assays were performed at 33°C in the presence of the indicated mGFP-TPX2 concentration and 10 µM tubulin. 373 pM γTuRC were used for immobilization. Representative TIRFM images of microtubules (magenta) nucleated in presence of γTuRC and 390 nM TPX2 (green) are shown in **(B)**. Nucleated microtubules were classified into 4 categories: (1) γTuRC-mediated nucleation (black line, first row of images), (2) microtubules nucleating from TPX2 (green line) are microtubules growing from TPX2 accumulations on the surface (second row of images) or TPX2 accumulations which land on the surface after forming a microtubule (third row of images), (3) spontaneously nucleated microtubules which grow aligned with an existing microtubule (fourth row of images) and (4) and spontaneously nucleated microtubules which elongate and diffuse on the surface (fifth row of images). Lines represent the linear regression. All error bars are s.e.m. For symbols without visible error bars, error bars are smaller than the symbol size. Field of view was always 164 µm x 164 µm. Fluorescence intensities are directly comparable. Scale bars as indicated. t=0 is 2 min after placing the sample at 33 °C.

Figure S5

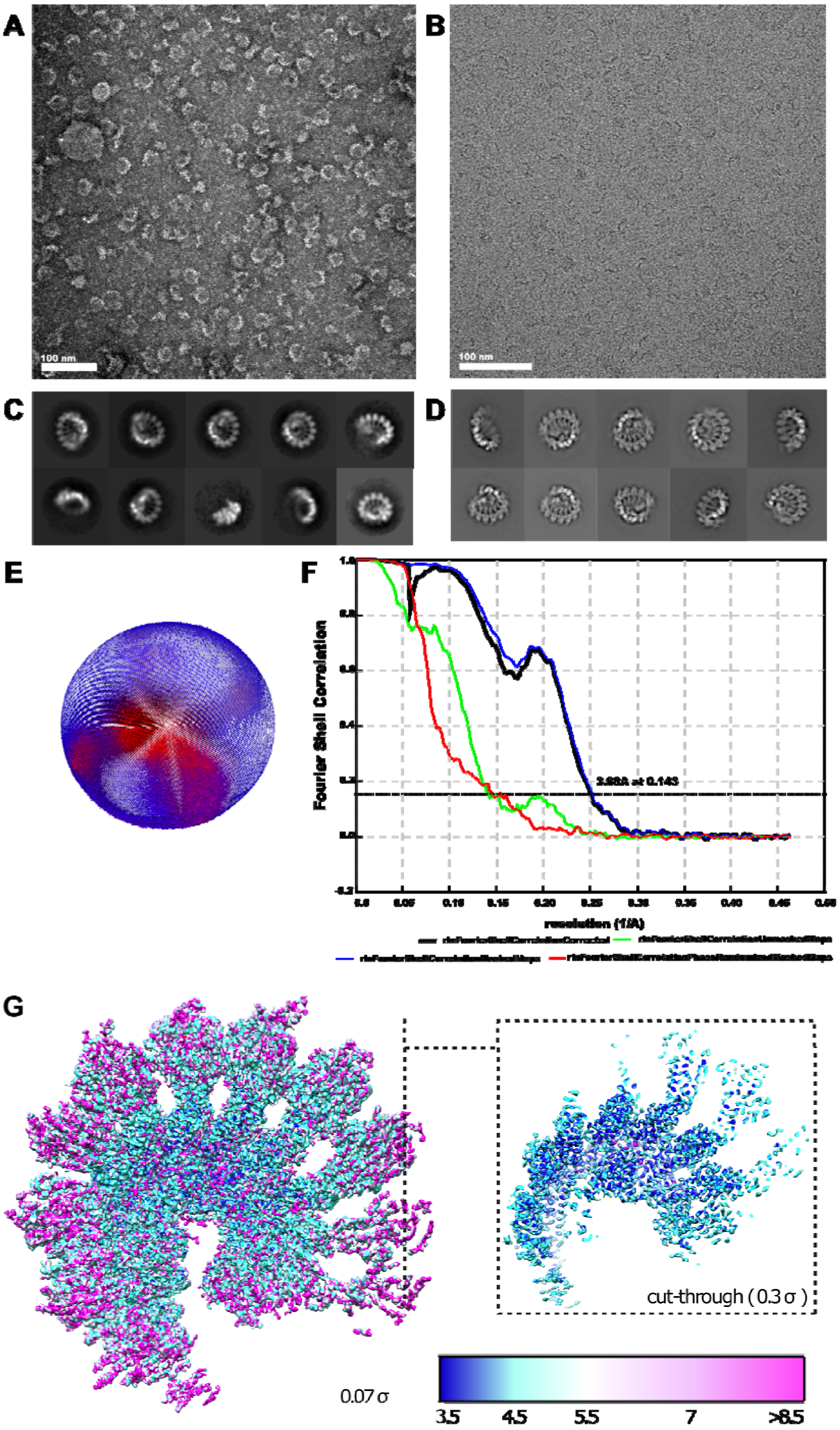

A
B
100 nm
100 nm
C
D
E
F
Fourier Shell Correlation
resolution (1/A)
0.0
0.05
0.10
0.15
0.20
0.25
0.30
0.35
0.40
0.45
0.50
G
cut-through (0.3 σ)
0.07 σ
3.5
4.5
5.5
7
>8.5

Figure S5. Electron microscopy image acquisition and processing (Related to Figs. 5 and 6). (A) Micrograph of a negatively stained sample. **(B)** Cryo-electron micrograph of a frozen-hydrated sample. **(C)** 2D class averages deriving from negative stain images. **(D)** 2D class averages deriving from cryo-EM images. **(E)** Angular distribution of particles used in the final cryo-EM reconstruction. **(F)** Fourier shell correlation indicating a resolution of 4 Å according to the 0.143 criterion. **(G)** Cryo-EM map colored according to the local resolution (after RELION3 postprocessing), as determined by using ResMap.

Figure S6

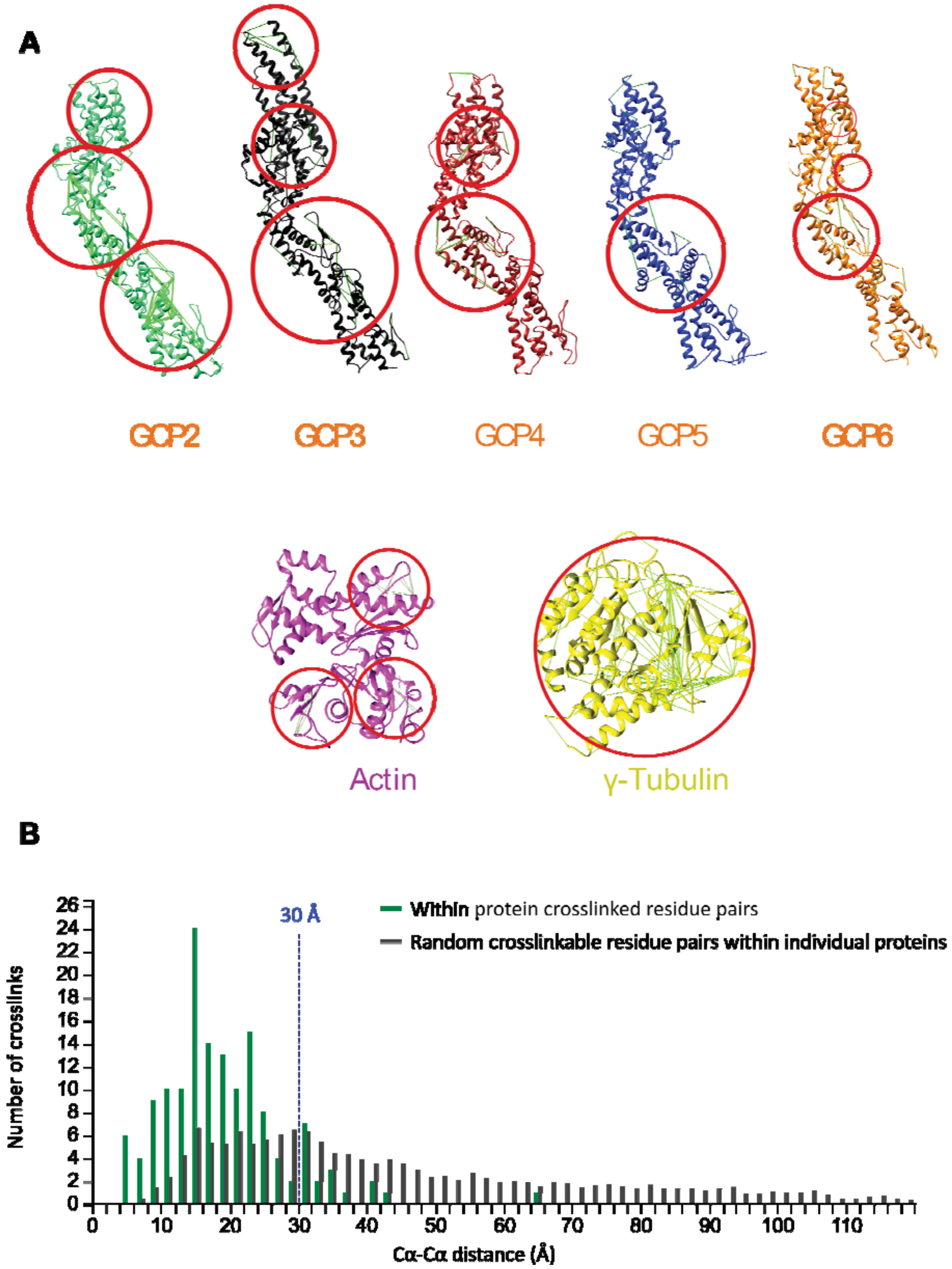

Figure S6. Summary of CLMS results (Related to Figs. 6 and 7). (A) Intramolecular crosslinks detected in CLMS experiments. Crosslinks are highlighted with a green silhouette. Red circles indicate clusters of crosslinks that measure 3 nm or shorter (compatible with the linker length of the BS3 crosslinker). **(B)** Distance distribution of within protein crosslinks detected in γTuRC complex (n=146). Theoretical crosslinking limit (30 Å) is indicated.
