# Supplementary material for: Microtubule Nucleation Properties of Single Human γTuRCs Explained by Their Cryo-EM Structure
Source: Dev Cell. 2020 Jun 8;53(5):603–617.e8. doi: 10.1016/j.devcel.2020.04.019 (PMC7280788; doi:10.1016/j.devcel.2020.04.019)
Supplement: Document S2. Article plus Supplemental Information [file mmc12.pdf]

Developmental Cell

Article

Microtubule Nucleation Properties of Single Human γTuRCs Explained by Their Cryo-EM Structure

Graphical Abstract

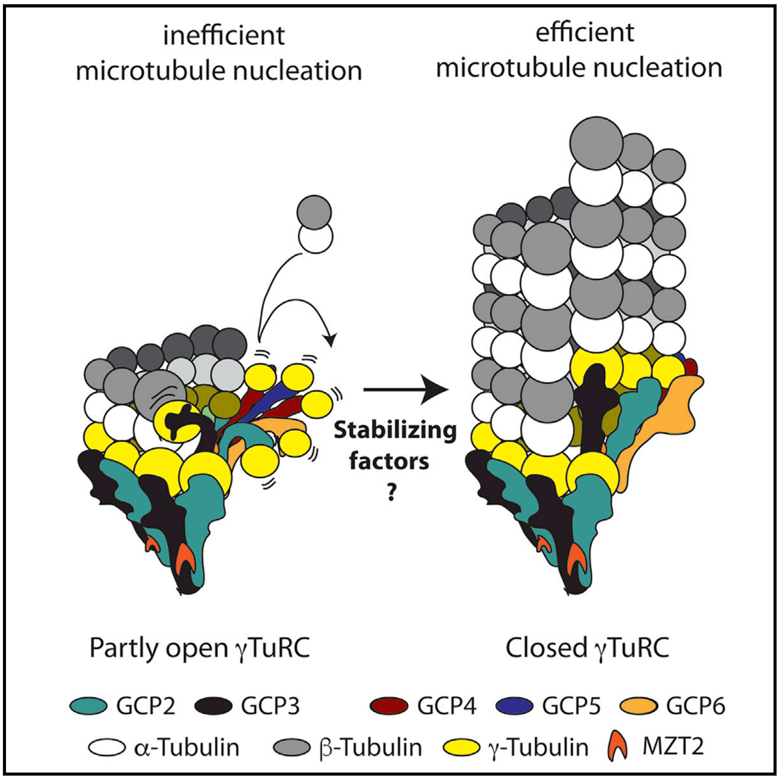

Authors

Tanja Consolati, Julia Locke, Johanna Roostalu, ..., Juri Rappsilber, Alessandro Costa, Thomas Surrey

Correspondence

alessandro.costa@crick.ac.uk (A.C.), thomas.surrey@crg.eu (T.S.)

In Brief

Consolati et al. find that microtubule nucleation by individual γTuRC complexes is inefficient despite its proposed role as a nucleating template. A 4 Å structure of the complex reveals a mismatch with the microtubule structure, explaining the inefficiency of nucleation and providing a possible mechanism for the regulation of nucleation.

Highlights

- The γ-tubulin ring complex (γTuRC) nucleates microtubules and caps their minus ends
- Microtubule nucleation from purified γTuRC is highly cooperative, yet inefficient
- A partly open, asymmetric structure of γTuRC explains inefficient nucleation
- Actin and MZT2 stabilize the closed part of the γTuRC structure

Consolati et al., 2020, Developmental Cell *53*, 603–617
June 8, 2020 © 2020 The Author(s). Published by Elsevier Inc.
https://doi.org/10.1016/j.devcel.2020.04.019

CellPress

Developmental Cell

CellPress
OPEN ACCESS

Article

Microtubule Nucleation Properties of Single Human γTuRCs Explained by Their Cryo-EM Structure

Tanja Consolati,[1,2,6] Julia Locke,[1,6] Johanna Roostalu,[1,7] Zhuo Angel Chen,[3,4] Julian Gannon,[1] Jayant Asthana,[1,2] Wei Ming Lim,[1,2] Fabrizio Martino,[1] Milos A. Cvetkovic,[1] Juri Rappsilber,[3,4] Alessandro Costa,[1,*] and Thomas Surrey[1,2,5,8,*]
[1]The Francis Crick Institute, 1 Midland Road, London NW1 1AT, UK
[2]Centre for Genomic Regulation (CRG), Barcelona Institute of Science and Technology (BIST), Dr Aiguader 88, 08003 Barcelona, Spain
[3]Bioanalytics, Institute of Biotechnology, Technische Universität Berlin, Berlin, Germany
[4]Wellcome Centre for Cell Biology, University of Edinburgh, Edinburgh, UK
[5]ICREA, Passeig de Lluis Companys 23, 08010 Barcelona, Spain
[6]These authors contributed equally
[7]Present address: The Wellcome Trust, London, United Kingdom
[8]Lead Contact
*Correspondence: alessandro.costa@crick.ac.uk (A.C.), thomas.surrey@crg.eu (T.S.)
https://doi.org/10.1016/j.devcel.2020.04.019

SUMMARY

The γ-tubulin ring complex (γTuRC) is the major microtubule nucleator in cells. The mechanism of its regulation is not understood. We purified human γTuRC and measured its nucleation properties in a total internal reflection fluorescence (TIRF) microscopy-based real-time nucleation assay. We find that γTuRC stably caps the minus ends of microtubules that it nucleates stochastically. Nucleation is inefficient compared with microtubule elongation. The 4 Å resolution cryoelectron microscopy (cryo-EM) structure of γTuRC, combined with crosslinking mass spectrometry analysis, reveals an asymmetric conformation with only part of the complex in a "closed" conformation matching the microtubule geometry. Actin in the core of the complex, and MZT2 at the outer perimeter of the closed part of γTuRC appear to stabilize the closed conformation. The opposite side of γTuRC is in an "open," nucleation-incompetent conformation, leading to a structural asymmetry explaining the low nucleation efficiency of purified human γTuRC. Our data suggest possible regulatory mechanisms for microtubule nucleation by γTuRC closure.

INTRODUCTION

Microtubule nucleation in cells is spatially and temporally controlled to ensure proper cytoskeleton function. The major nucleator is the γ-tubulin ring complex (γTuRC) in which several γ-tubulin complex proteins (GCPs) arrange 14 γ-tubulins into a helical arrangement so that they can serve as a template for microtubule nucleation (Kollman et al., 2011; Tovey and Conduit, 2018). The structure of γTuRC is best understood in budding yeast where 7 smaller "Y-shaped" γTuSC complexes, each consisting of 2 γ-tubulins and one copy of GCP2 and GCP3, assemble into a conically shaped assembly upon recruitment to the spindle pole body by Spc110 (Kollman et al., 2010). A cryoelectron microscopy (cryo-EM) reconstruction of budding yeast γTuSC in complex with a Spc110 fragment at 8-Å resolution revealed gaps between every second γ-tubulin in γTuRC creating a mismatch with the microtubule structure (Kollman et al., 2015). Microtubule nucleation by budding yeast γTuRC in this "open" conformation could be improved 2–3-fold by artificially closing these gaps through chemical crosslinking, suggesting a possible mechanism for activation of nucleation by budding yeast γTuRC (Kollman et al., 2015).

In fission yeast, filamentous fungi, and metazoans, some GCP2s and GCP3s are replaced in the complex by additional GCP4, GCP5, and GCP6 proteins and in metazoans γTuRC is a stable complex whose assembly is independent of the recruitment to target structures, such as centrosomes (Farache et al., 2018; Lin et al., 2015; Murphy et al., 2001; Oegema et al., 1999; Tovey and Conduit, 2018). The exact stoichiometry and subunit order of human γTuRC is not known. Several proteins have been implicated in activating γTuRC, among which are MZT1 and MZT2, also sometimes classified as core components of the metazoan complex (Hutchins et al., 2010; Kollman et al., 2011; Lin et al., 2016; Teixidó-Travesa et al., 2012), the recruitment factors CDK5Rap2 (functional homolog of budding yeast Spc110) (Choi et al., 2010; Lin et al., 2014; Muroyama et al., 2016) and NEDD1 (Scrofani et al., 2015), or microtubule dynamics regulators, such as the microtubule polymerase chTOG (XMAP215) or the multifunctional, catastrophe-suppressing protein TPX2 (Alfaro-Aco et al., 2017; Popov et al., 2002; Scrofani et al., 2015; Thawani et al., 2018).

A clear understanding of the mechanisms by which the efficiency of microtubule nucleation by human γTuRC is regulated is however lacking. The kinetics of microtubule nucleation either

This is an open access article under the CC BY license (http://creativecommons.org/licenses/by/4.0/).

in the absence or presence of purified γTuRC have typically been measured either by following the turbidity of suspensions of nucleating microtubules over time or by fluorescence microscopy imaging of microtubules at distinct times after mixing γTuRC with tubulin (Oegema et al., 1999; Voter and Erickson, 1984). Both types of assays have disadvantages, as they cannot distinguish easily between γTuRC-mediated and spontaneous microtubule nucleation and between microtubule nucleation and effects produced by microtubule growth and/or dynamic instability.

To overcome these limitations, we developed a microscopy-based *in vitro* nucleation assay that allows the real-time observation of the nucleation of individual microtubules by single surface-immobilized human γTuRCs, and we studied the structure of the γTuRC complex by cryo-EM combined with crosslinking mass spectrometry (CLMS). We found that human γTuRC-mediated nucleation is stochastic, highly cooperative, and faces a significant kinetic barrier. γTuRC improved the nucleation efficiency compared with spontaneous microtubule nucleation, but templated nucleation was still less efficient than microtubule plus-end elongation. A 4-Å resolution structure of human γTuRC revealed several features that are distinctly different from the structure of budding yeast γTuRC; surprisingly, γTuRC harbors actin in its central core and the complex is markedly asymmetric only partially matching the geometry of the active form of yeast γTuRC. Our results provide an explanation for the observed kinetic barrier for nucleation by human γTuRC and suggest a possible stimulatory function of additional factors that would morph γTuRC into a fully activated form.

RESULTS

Purification of Biotinylated Human γTuRC

We generated a HeLa Kyoto cell line that expressed a biotin acceptor peptide (BAP) and monomeric blue fluorescence protein (mBFP)-tagged GCP2 from a randomly integrated gene. Tagged GCP2 became incorporated into the human γTuRC complex and was biotinylated without compromising γTuRC function as indicated by the correct localization of the fluorescent complex to centrosomes and normal cell growth. We purified ~ 0.1 mg tagged γTuRC from 120 g of cells in a one-day procedure using anion exchange, biotin affinity, and size exclusion chromatography (STAR Methods) (Figures 1A, 1B, and S1).

Using mass spectroscopy, we identified all human γTuRC core subunits (γ-tubulin, GCP2-6), as well as MZT1, MZT2, and actin, which were co-purified in previous γTuRC purifications (Figure S1C; Data S1) (Choi et al., 2010; Hutchins et al., 2010; Oegema et al., 1999; Teixidó-Travesa et al., 2012; Thawani et al., 2018). Additionally, we verified most subunits also by western blot using specific antibodies (Figure 1C; see also Figure S1B), indicating that the human γTuRC complex was successfully purified using our biotin affinity purification strategy. Purified γTuRC appeared as the typical characteristic "rings" with ~25-nm diameter in negative-stain electron microscopy images (Figure 1D) (Zheng et al., 1995), confirming the presence of properly assembled complexes.

γTuRC Nucleates Single Microtubules and Caps Their Minus Ends

Next, we set up a microscopy-based real-time γTuRC-mediated microtubule nucleation assay (Figure 1E). We used NeutrAvidin to immobilize purified biotinylated γTuRC on a biotin-polyethylene glycol (PEG)-functionalized glass surface. Specific immobilization of biotinylated and mBFP-tagged γTuRC could be verified by measuring the mBFP fluorescence on NeutrAvidin surfaces and on surfaces lacking NeutrAvidin using total internal reflection fluorescence (TIRF) microscopy (Figures 1F and S2A). In the presence of CF640R-labeled tubulin, microtubules nucleated from the γTuRC-coated surface, whereas hardly any microtubules nucleated in the absence of γTuRC under these conditions (Figures 1F and 1G).

Only one end of γTuRC-nucleated microtubules grew, whereas the other was tethered to the surface likely via γTuRC (Figures 2A, top rows, and 2B, left; Video S1). In contrast, when microtubules elongated from surface-immobilized, stabilized microtubule "seeds" in control experiments, both microtubule ends grew (Figures 2A, bottom row, and 2B, right) with the faster plus-end growth speed of 26.8 ± 0.4 nm/s essentially equating the growth speed of γTuRC-nucleated microtubules of 26.3 ± 0.4 nm/s (errors are SEM) (Figure 2C, top). This observation demonstrates that γTuRC-nucleated microtubules grow exclusively at their plus end. Minus ends grew from "seeds" with a speed of 7.0 ± 0.2 nm/s, whereas surface-anchored minus ends of γTuRC-nucleated microtubules did not grow (Figure 2C, bottom). mGFP-labeled growing microtubule end marker EB3 decorated only the growing plus ends of γTuRC-nucleated microtubules (Figures 2D and 2E; Video S2), similar to the situation in the cell (Akhmanova and Steinmetz, 2015). In rare cases, γTuRC-nucleated microtubules first grew out of the TIRF field and later, remaining γTuRC-anchored, aligned with the surface only growing with one end (Figures S2Bi and S2Bii). These data clearly demonstrate that, at our experimental conditions, plus ends of γTuRC-nucleated microtubules are dynamic and minus ends are capped by γTuRC. The occasional microtubule that nucleated in solution and then landed on the glass surface was easily distinguished from γTuRC-nucleated microtubules, as it became suddenly visible as an already elongated microtubule, displaying two dynamic microtubule ends and often also diffusing along the surface (Figures S2Biii and S2Biv).

Imaging the mBFP fluorescence of single immobilized γTuRC complexes at a reduced γTuRC density revealed that all surface-nucleated microtubules originated from a mBFP-labeled γTuRC (Figures 2F and 2G; Video S3). Taken together, these data demonstrate that immobilized γTuRC stimulates microtubule nucleation, generating microtubules with a capped minus and a dynamic plus end. We did not observe microtubule detachment from immobilized γTuRC, indicating that γTuRC is stably bound to its nucleated microtubule within the entire duration of our experiments (20 min).

γTuRC-Mediated Microtubule Nucleation Is Stochastic, Cooperative, and Not Very Efficient

Next, we quantified the number of γTuRC-nucleated microtubules per field of view, excluding the small fraction of microtubules nucleated in solution. Counting the γTuRC-nucleated microtubules, showed a linear increase of their number with time (Figures 3A and 3Bi; Video S4). This demonstrates that γTuRC-mediated nucleation is a stochastic process with constant nucleation probability. Increasing the γTuRC density on the surface while keeping the tubulin concentration constant at 15 μM, demonstrated that the overall nucleation rate (increase of

CellPress
OPEN ACCESS

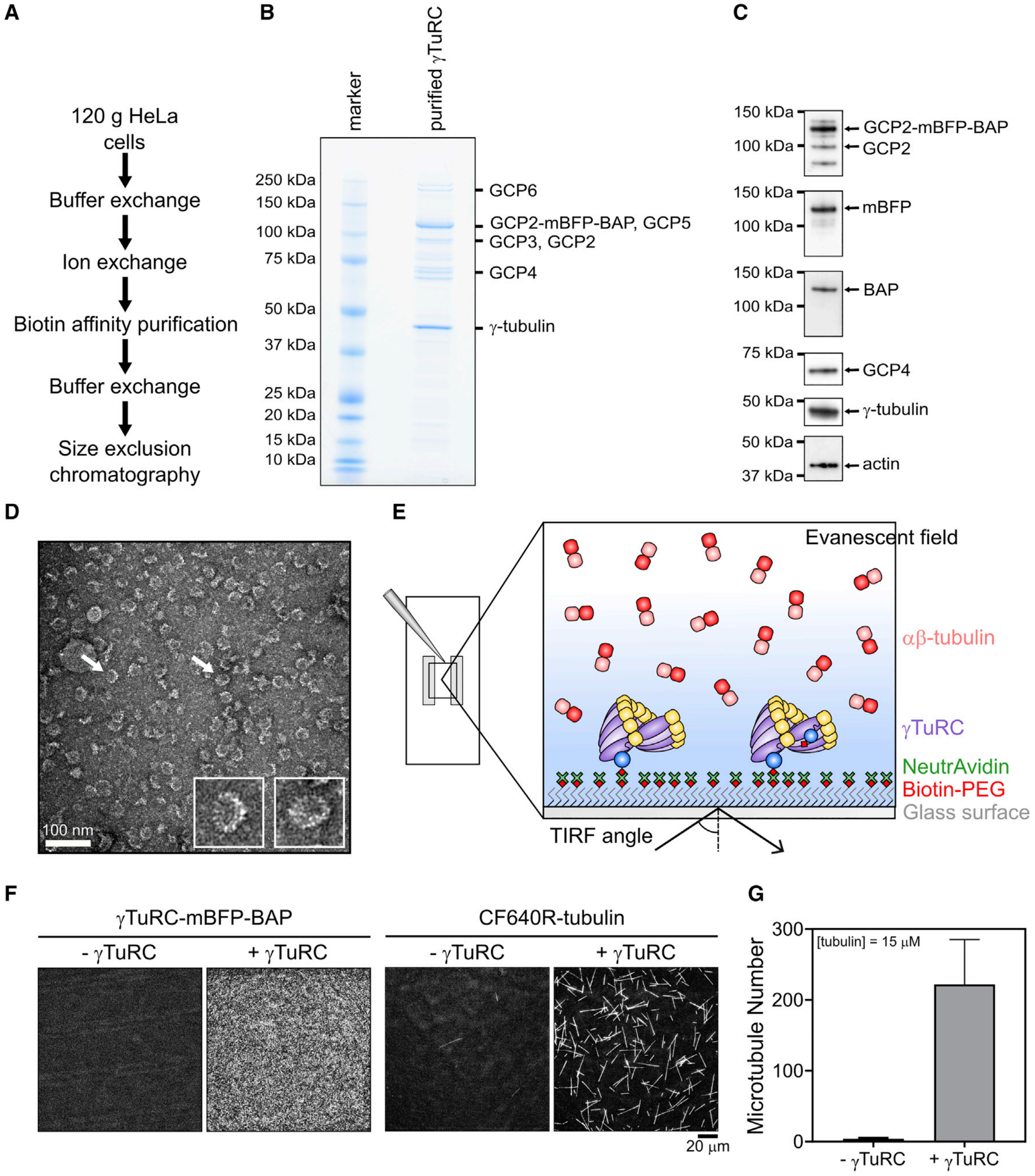

Figure 1. Purification and Characterization of γTuRC-mBFP-BAP

(A) Overview of purification steps.

(B) Coomassie-stained SDS-PAGE of purified γTuRC. Protein bands corresponding to γTuRC subunits as identified by mass spectrometry are labeled.

(C) Western blots of purified γTuRC using antibodies against γ-tubulin, GCP2, GCP4, actin, and mBFP. Biotinylation of the BAP was assessed by immunoblotting using horse radish peroxidase (HRP)-coupled streptavidin.

(D) Negative-stain electron microscopy of purified γTuRC showing the expected 25-nm diameter ring structures. Two examples (white arrows) are shown as insets at higher magnification.

(E) Schematic of TIRFM-based real-time γTuRC nucleation assay. Biotinylated fluorescent γTuRC is immobilized on a biotin-PEG-functionalized glass surface via NeutrAvidin. αβ-tubulin is added to initiate microtubule nucleation by immobilized γTuRC.

(F) Representative TIRFM images showing the mBFP channel to visualize γTuRC on the surface (left panel) and showing the CF640R-tubulin channel to visualize nucleated microtubules (right panel) at t = 20 min after start of microtubule nucleation by a temperature jump to 33°C. 373 pM γTuRC was used for immobilization and the final CF640R-tubulin concentration was 15 μM. A representative control at 15 μM CF640R-tubulin without γTuRC is also shown. Intensities in the images are directly comparable.

(G) Bar graph of the average microtubule number nucleated by surface-immobilized γTuRC (373 pM used for immobilization) within 15 min in presence of 15 μM CF640R-tubulin (n = 3). Error bars are SD. Scale bars as indicated. t = 0 is 2 min after placing the sample at 33°C. See also Figures S1 and S2A.

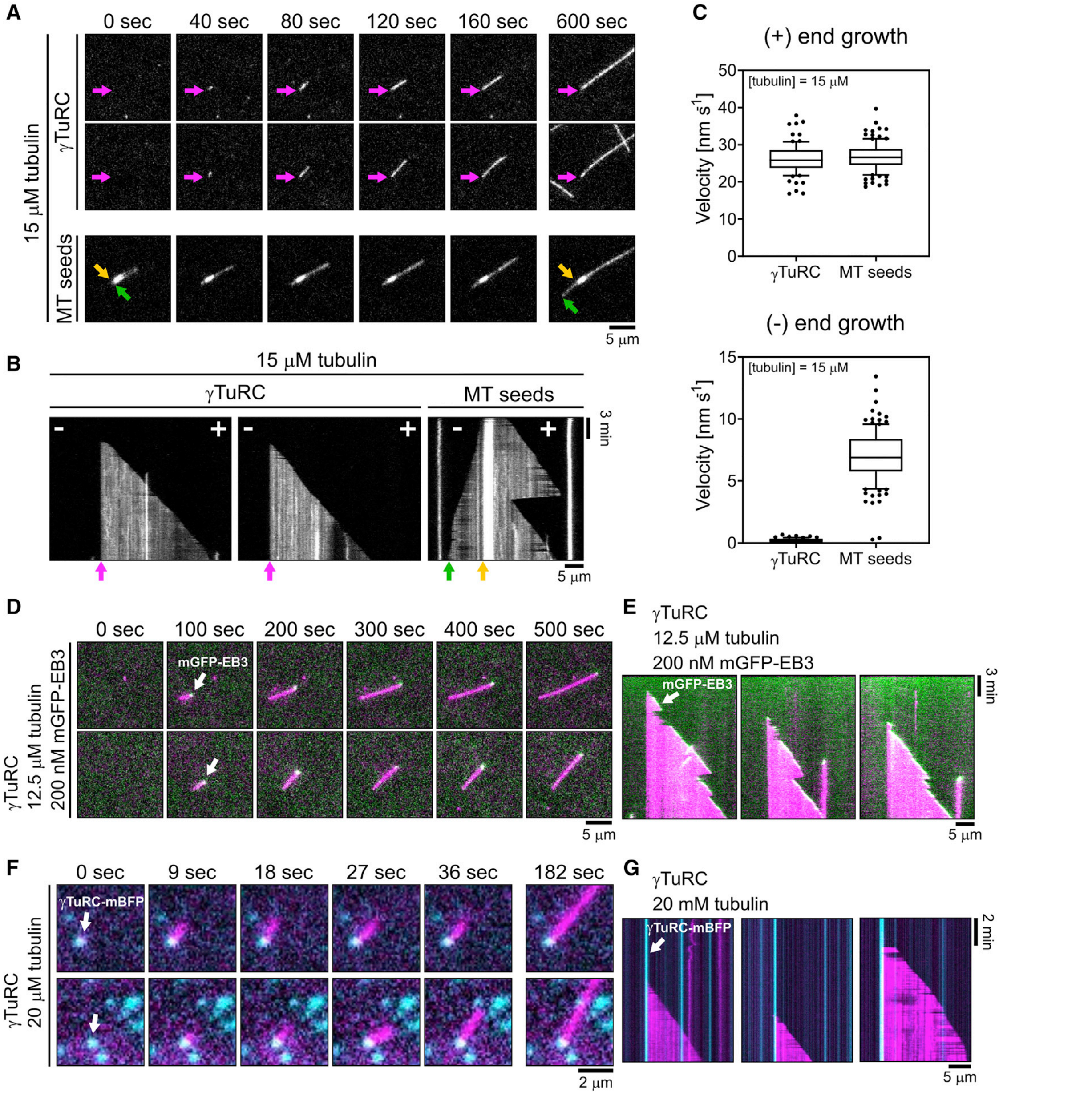

Figure 2. γTuRC Nucleates and Caps Microtubules at Their Minus End

(A–C) Comparison between γTuRC microtubule nucleation assay and microtubule seed assay. Both assays were performed in the presence of 15 μM CF640R-tubulin. For γTuRC microtubule nucleation assay 373 pM γTuRC were used for immobilization. (A) Representative time series of individual microtubules (2 top rows of panels) nucleated on a γTuRC surface showing a static (purple arrow) and a fast-growing microtubule end. A control without γTuRC (bottom row) shows a microtubule growing from a stabilized microtubule "seed," displaying two growing microtubule ends with the minus end (purple arrow) growing more slowly than the plus end. (B) Representative TIRFM kymographs of microtubules nucleated by surface-immobilized γTuRC. For comparison a kymograph of a microtubule grown from a microtubule seed at the same tubulin concentration is shown. (C) Box-and-whiskers plots of microtubule plus-end (top) and minus-end (bottom) growth speeds for γTuRC nucleation assays and microtubule seed assays.

(D and E) mGFP-EB3 tracks the growing plus end of γTuRC nucleated microtubules. Assays were performed in the presence of 12.5 μM CF640R-tubulin and 200 nM mGFP-EB3 using 373 pM γTuRC for immobilization. Data were pooled from two independent experiments. Number of microtubule growth speeds measured per conditions: γTuRC nucleation assay, plus-end growth: n = 86, minus-end growth: n = 71; microtubule seed assay, plus-end growth: n = 110, minus-end growth: n = 123. For the box-and-whiskers plots, boxes range from 25th to 75th percentile, the whiskers span from 10th to 90th percentile, and the horizontal line marks the mean value. (D) Representative time series of merged TIRFM images of two individual microtubules (magenta) nucleated from a γTuRC surface. mGFP-EB3 (green, white arrow) tracks the growing microtubule plus end, while the microtubule minus end is static. (E) Corresponding TIRFM kymographs.

(F and G) Microtubules are nucleated by single γTuRC molecules. Assays were performed in the presence of 20 μM CF640R-tubulin using 27 pM γTuRC for immobilization. (F) Representative time series of merged TIRFM images showing individual microtubules (magenta) nucleated from single immobilized γTuRC molecules (cyan, white arrow). (G) Corresponding TIRFM kymographs. All experiments were performed at 33°C. Scale bars as indicated. t = 0 is 2 min after placing the sample at 33°C. See also Figure S2B; Videos S1–S3.

CellPress
OPEN ACCESS

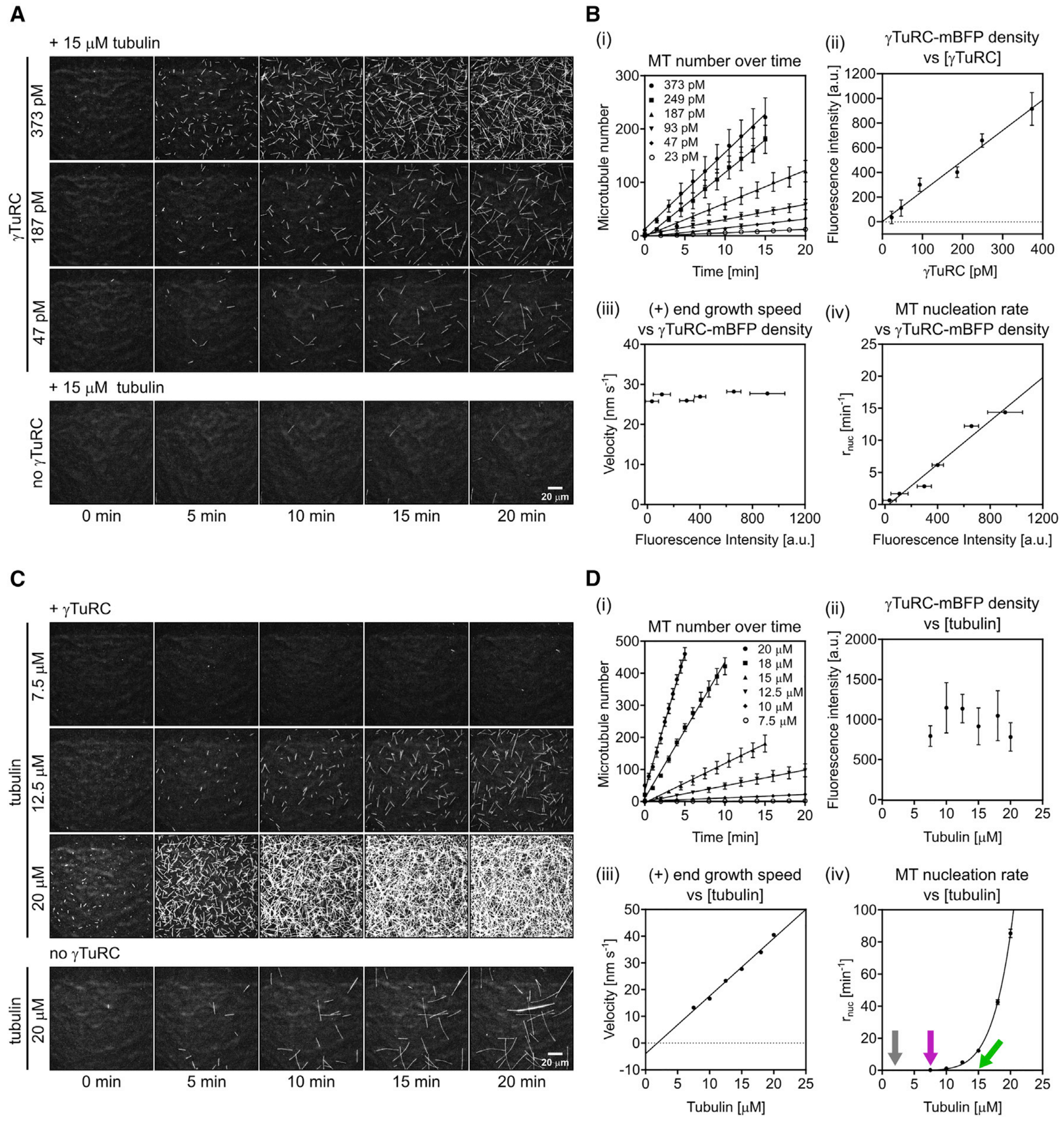

Figure 3. The Microtubule Nucleation Efficiency of γTuRC Depends on γTuRC Surface Density and Tubulin Concentration

(A and B) Microtubule nucleation at 33°C in the presence of 15 μM CF640R-tubulin at varying γTuRC concentrations used for immobilization (23, 47, 93, 187, 249, and 373 pM). (A) Representative time series of TIRFM images at the indicated γTuRC concentrations (top panel). For comparison, spontaneous microtubule nucleation in the absence of γTuRC at the same tubulin concentration is shown (bottom panel). (B) Plots showing (i) a linear increase in microtubule number over time, (ii) the mean γTuRC surface density (mBFP fluorescence in the field of view), (iii) the mean microtubule plus-end growth speed, and (iv) the mean microtubule nucleation rate (bottom right) at different γTuRC concentrations. Number of microtubule growth speeds measured per conditions: 23 pM, n = 27; 47 pM, n = 64; 93 pM, n = 96; 187 pM, n = 191; 249 pM, n = 160; 373 pM, n = 302.

(C and D) Microtubule nucleation in presence of 373 pM γTuRC used for immobilization at varying CF640R-tubulin concentrations (7.5, 10, 12.5, 15, 18, and 20 μM). (C) Representative time series of TIRFM images of microtubule nucleation in the presence of the indicated CF640R-tubulin concentrations (top panel). For comparison, spontaneous microtubule nucleation in the absence of γTuRC is shown for the highest tested tubulin concentration (20 μM) (bottom panel). Spontaneous microtubule nucleation was always much less than γTuRC-mediated nucleation comparing the same tubulin concentrations (not shown). (D) Plots showing (i) the linear increase in microtubule number over time, (ii) the mean γTuRC surface density (mBFP fluorescence in the field of view), (iii) the mean microtubule plus-end growth speed, and (iv) the mean microtubule nucleation rate at different tubulin concentrations. Arrows mark the critical tubulin concentration for microtubule elongation (gray) defined as the intercept of the fit in Figure 3Diii with the x axis and the minimal concentration required for γTuRC-mediated nucleation (purple) and for spontaneous nucleation in the absence of γTuRC (green) both defined empirically as the tubulin concentration at which on average 1 or more microtubules become visible within 20 min in the field of view (164 × 164 μm). Number of microtubule growth speeds measured per conditions: 7.5 μM, n = 9; 10 μM, n = 51; 12.5 μM, n = 210; 15 μM, n = 190; 18 μM, n = 237; 20 μM, n = 244. Data for plots were pooled from at least three independent experiments. The plot of the nucleation rate against tubulin concentration (see Figure 3Div) was fit using a power law function. All other lines represent a linear

(legend continued on next page)

microtubule number per time and per surface area) increased with the γTuRC concentration used for γTuRC surface immobilization (Figures 3A and 3Bii; Video S4) and with the measured mBFP intensity at the surface, i.e., the γTuRC density (Figure 3Biv). The microtubule growth speed was unaffected by the γTuRC density (Figure 3Biii), in agreement with the tubulin concentration essentially remaining unchanged in these experiments. We conclude that γTuRC stimulates nucleation in a dose-dependent manner.

Next, we changed the tubulin concentration keeping the γTuRC density constant (Figures 3C and 3Dii; Video S5). While the microtubule growth speed increased linearly with tubulin concentration, as expected (Figure 3Diii), the nucleation rate increased very non-linearly with tubulin concentration (Figures 3C, 3Di, and 3Div), in agreement with an early study using *Xenopus* γTuRC (Zheng et al., 1995). A fit to the γTuRC-mediated dependence of the nucleation rate on the tubulin concentration using a power law (line in Figure 3Div) yielded an exponent of 6.7, demonstrating that γTuRC-mediated nucleation is a highly cooperative process, similar to spontaneous nucleation in solution (Erickson and Pantaloni, 1981; Kuchnir Fygenson et al., 1995; Voter and Erickson, 1984); however, γTuRC-mediated nucleation is clearly more efficient than spontaneous nucleation at all tubulin concentrations (Figure S2C). The fit to the power law can also be used to provide an estimate for the minimal size of a templated tubulin assembly on γTuRC that allows stable microtubule outgrowth, which is in the range of one to two times the value of the exponent (Kuchnir Fygenson et al., 1995; Voter and Erickson, 1984). The minimal tubulin concentration required for detectable γTuRC-mediated nucleation was 7.5 μM and hence significantly lower than the ~15 μM for spontaneous nucleation in solution in the absence of γTuRC (Figure 3Div: microtubule elongation [gray arrow], γTuRC-mediated nucleation [purple arrow], and spontaneous nucleation [green arrow]) (Figure S2C) (Brouhard and Rice, 2018; Gard and Kirschner, 1987a; Roostalu and Surrey, 2017). However, γTuRC-mediated nucleation required a tubulin concentration still higher than the 2 μM tubulin threshold above which pre-existing microtubule plus ends elongate (Figure 3Diii) (Voter and Erickson, 1984; Wieczorek et al., 2015). This indicates that templating a new microtubule from γTuRC is easier than forming a new microtubule in solution, but it is clearly less efficient compared with elongating an existing growing microtubule end.

Comparing the number of γTuRC complexes on the surface as measured by the number of mBFP dots with the number of nucleation events at 20 μM tubulin revealed that only ~0.5% of complexes nucleated a microtubule within 9 min at our conditions. Therefore, γTuRC-mediated microtubule nucleation appears to be rather inefficient, suggesting that most likely additional factors are required for activating the complex or for promoting nucleation by stabilizing a freshly nucleated nascent microtubule. Therefore, using our nucleation assay we tested the effects on human γTuRC-mediated microtubule nucleation elicited by three proteins that are known to affect microtubule dynamics by preferentially binding to microtubule ends and that have also been reported to affect nucleation in different ways.

chTOG and TPX2 Stimulate γTuRC-Mediated Microtubule Nucleation

The microtubule polymerase chTOG/XMAP215 (Brouhard et al., 2008; Gard and Kirschner, 1987b) is known to mildly stimulate spontaneous microtubule nucleation *in vitro* (Ghosh et al., 2013; Roostalu et al., 2015). In the presence of human chTOG, nucleation from surface-immobilized γTuRC is strongly promoted (Figures 4A–4C; Video S6), in agreement with previous reports using the budding yeast and *Xenopus* orthologs of these proteins (Gunzelmann et al., 2018; Thawani et al., 2018). We find here that chTOG stimulates microtubule nucleation by human γTuRC by a factor of up to 21-fold (Figure 4C), with the stimulatory effect saturating in a physiological concentration range (100 nM in *Xenopus* egg extract; Kronja et al., 2009; Reber et al., 2013; Wühr et al., 2014). Saturation of the acceleration of microtubule plus-end growth by chTOG occurs at a similar concentration (Figures 4D and S3), suggesting that both chTOG effects are related and saturate when plus-end-binding sites at microtubule ends are fully occupied by chTOG. Thus, acceleration of outgrowth of a nascent microtubule forming on γTuRC may be one mechanism to increase overall nucleation efficiency (Roostalu and Surrey, 2017).

TPX2, a protein involved in chromatin-dependent microtubule nucleation, can also stimulate nucleation *in vitro* (Alfaro-Aco et al., 2017; Roostalu et al., 2015; Schatz et al., 2003). We tested here to which extent TPX2 could stimulate microtubule nucleation from immobilized γTuRC. We measured microtubules nucleating from the γTuRC surface, excluding the only minor fraction of those microtubules that nucleate from local TPX2 accumulations possibly representing recently reported TPX2 condensates forming at high TPX2 concentrations (King and Petry, 2020) (Figure S4). We observed that TPX2 stimulated γTuRC-mediated microtubule nucleation in a dose-dependent manner (Figures 4E–4G; Video S7), however, only at rather high concentrations compared with physiological TPX2 concentrations (25–100 nM in *Xenopus* egg extract; Gruss et al., 2001; Thawani et al., 2019; Wühr et al., 2014). TPX2 had no strong effect on microtubule growth speed (Figure 4H), as reported previously (Roostalu et al., 2015; Wieczorek et al., 2015). Therefore, TPX2 likely promotes γTuRC-mediated nucleation *in vitro* by a different mechanism compared with chTOG, possibly by suppressing depolymerization of a nascent microtubule on γTuRC, through its catastrophe-suppressing activity (Roostalu et al., 2015; Wieczorek et al., 2015). In contrast to chTOG and TPX2, we did not observe any effect of the plus-end-tracking protein EB3 on γTuRC-mediated nucleation (Figures 4I–4L).

The Cryo-EM Structure and CLMS Analysis of Human γTuRC

To understand the molecular basis of microtubule nucleation, we determined the cryo-EM structure of γTuRC to a resolution of 4 Å

regression. Nucleation rates (r_{nuc}) were taken from the slope of the linear regression of the increase of microtubule number over time (see Figures 3Bi and 3Di). All experiments were performed at 33°C. All error bars are SEM. For symbols without visible error bars, error bars are smaller than the symbol size. Field of view was always 164 × 164 μm. AU, arbitrary units. Fluorescence intensities are directly comparable. Scale bars as indicated. t = 0 is 2 min after placing the sample at 33°C. See also Figure S2C; Videos S4 and S5.

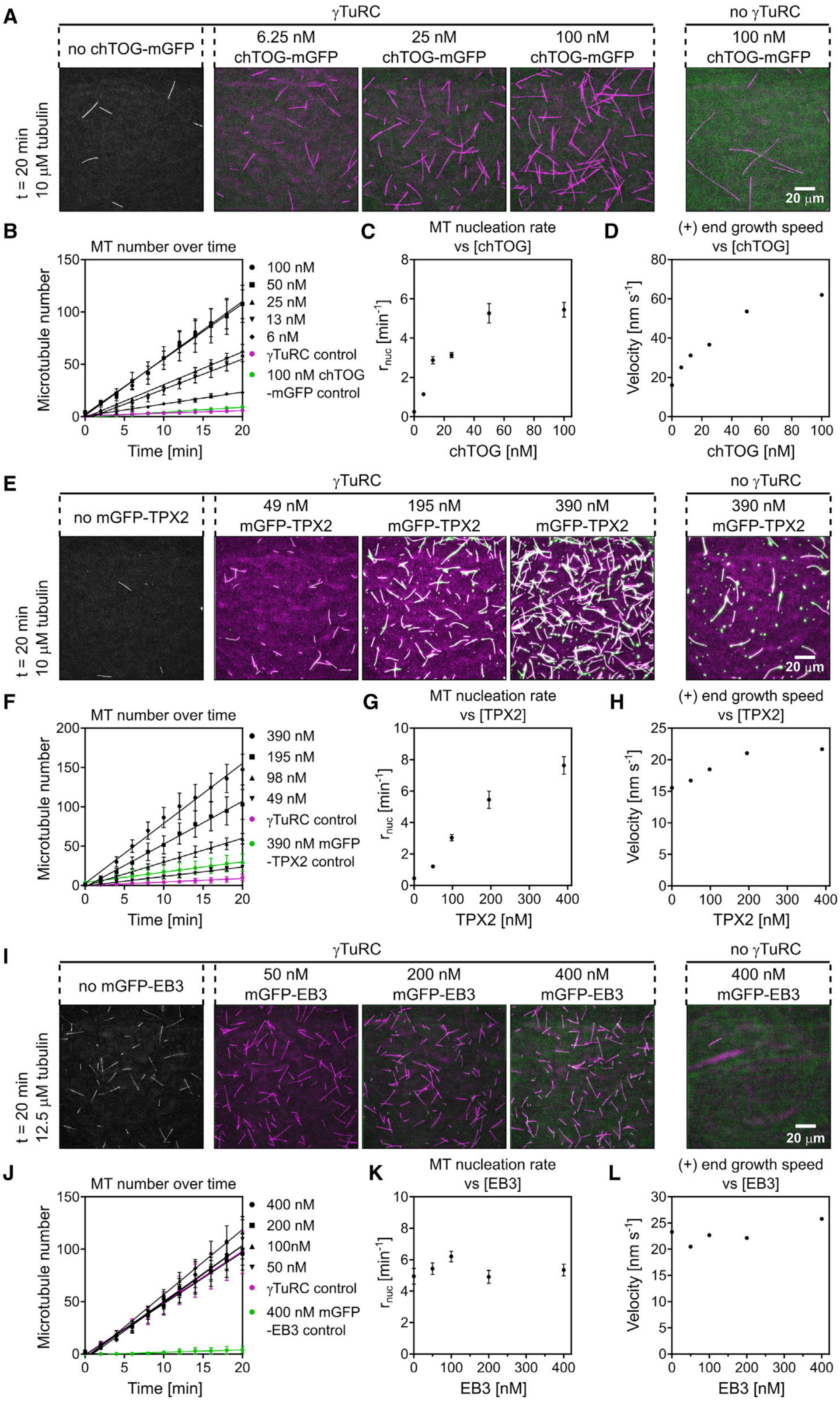

Figure 4. Microtubule Associated Proteins Can Increase the Microtubule Nucleation Efficiency of γTuRC

(A–D) γTuRC-mediated microtubule nucleation in the presence of different chTOG-mGFP concentrations (6, 13, 25, 50, and 100 nM). Assays were performed in the presence of chTOG-mGFP and 10 μM CF640R-tubulin using 373 pM γTuRC for immobilization. (A) Representative TIRFM images of γTuRC-mediated

(legend continued on next page)

(or 3.7 Å after cryo-EM density modification; Terwilliger et al., 2019; Figure S5; also see Table 1). The complex is arranged in a left-handed spiral cone (reminiscent of a churros paper wrap), narrowing at one end, with a 300-Å largest diameter and a height of 200 Å (Figure 5A). The spiral is formed by 14 similar modules ("stalks"), which support 14 globular features decorating the largest face of the complex. Comparison with the crystal structure of the GCP4 subunit of γTuRC (PDB:3RIP) (Guillet et al., 2011) allows the immediate identification of 14 different GCP protomers forming the spiral, which we number starting from the narrow bottom to the large-face top of the spiral. Notably, subunit 14 at the top of the spiral aligns with the lowermost subunit 1 (Figure 5B). Inter-GCP interactions closely resemble those observed at the GCP2-GCP3 interface visible in the yeast γTuSC complex structure (Kollman et al., 2015). While GCP subunits 1–8 in γTuRC engage in tight inter-protomer interactions that involve both the constricted side and the larger side of the cone, subunits 9–14 merely interact at the tip of the cone and appear more flexible as they depart radially from the core of the structure (Figure 5A). The globular densities decorating the wider side of the GCP cone was rigid-body fitted by 14 γ-tubulin protomers (PDB:1Z5W) (Aldaz et al., 2005), resulting in a configuration akin to the γTuSC structure (Kollman et al., 2015) (Figure 5B).

Although the local resolution of the γ-tubulin subunits varies around the ring, nucleotide density can clearly be observed when the local resolution is high enough, for example, for γ-tubulin in position 11 (Figure 5C). Densities appear less defined for γ-tubulin in particular for positions 12, 13, and 14. Our resolution does not allow discrimination between guanosine triphosphate (GTP) and guanosine diphosphate (GDP) in the active site. GCP2-interacting γ-tubulin from the N-terminal bottom of the spiral appears engaged with the GCP3-interacting γ-tubulin pointing toward the C-terminal top of the spiral. Conversely, gaps of varying extents can be identified between γ-tubulins engaged by other GCP subunits (Figure 5B). Due to limits in resolution, we cannot comment on differences in the configuration of lateral loops in the γ-tubulin subunits around the γTuRC spiral.

As our mass spectrometry analysis of the purified human γTuRC identified all five paralogous GCP2, 3, 4, 5, and 6 subunits (Figure S1C; Data S1), we sought to identify each subunit in our 14-mer complex. We first generated homology models for human GCP2 and GCP3 based on human GCP4. Although the two homology models appeared similar to each other, GCP3 presented a characteristic helical extension in the C-terminal, γ-tubulin-interacting domain (also known as GRIP2 domain; Guillet et al., 2011; Gunawardane et al., 2000; Murphy et al., 2001) (Figure 6A). Given their structural homology, unique, structured sequence insertion and the well-documented ability to heterodimerize, we built a dimeric model for GCP2-GCP3 and docked it around all possible positions within the γTuRC cone. We found that GCP2-GCP3 best fits GCP positions 1–2, 3–4, 5–6, 7–8, and 13–14 with cross-correlation scores of 0.71, 0.69, 0.70, 0.70, and 0.72, respectively (Figure 6A). Conversely, positions 9-10 and 11-12 scored poorly (0.43 and 0.40, respectively), indicating that these protomers likely contain GCP4, 5, and 6.

To locate GCP4 in GCP positions 9, 10, 11, and 12 of the cryo-EM map, we employed cross-correlation searches in the constricted N-terminal region of the GCP spiral, where local resolution ranges from 3 to 3.5 Å. The human GCP4 N-terminal domain ("GRIP1" domain, Guillet et al., 2011) extracted from the crystal structure showed the highest correlation at GCP in positions 9 (0.62) and 11 (0.58), whereas positions 10 and 11 yielded lower scores (0.35 and 0.41, respectively). Amino acidic side chains of alpha helices in the crystallographic model match the density features in the N-terminal GCP4 cryo-EM map without the need of any real-space refinement (Figure 6B), providing us with confidence in the subunit assignment. The rest of the atomic structure was split in two additional domains (middle, "MID", and "C-terminal"), which were docked as independent rigid bodies to achieve the best-fitting results (Figure 6B).

To validate our homology-model-based assignment and locate GCP subunits 5 and 6, we performed CLMS analysis on the purified γTuRC complex. Structural interpretation of inter-protein crosslinks was focused on protein pairs that were crosslinked with three or more residue pairs. 16 protein pairs including all 5 displayed in Figure 6C passed this cutoff. A strong BS3 crosslink signal can be observed between GCP2 and GCP3, supporting the notion that the GCP2-GCP3 heterodimer is found in multiple copies around the ring. GCP4 cross linked with GCP5 and GCP6, while GCP6 also was observed to interact with GCP2 and GCP3

microtubule nucleation in the presence of different chTOG-mGFP concentrations, as indicated. Microtubules are magenta, chTOG-mGFP is green. Plots showing, (B) linearly increasing microtubule numbers over time, (C) the mean microtubule nucleation rate, and (D) the mean microtubule growth speed at different chTOG-mGFP concentrations. Number of microtubule growth speeds measured per conditions: 23 pM, n = 27; 47 pM, n = 64; 93 pM, n = 96; 187 pM, n = 191; 249 pM, n = 160; 373 pM, n = 302.

(E–H) γTuRC-mediated microtubule nucleation in the presence of different mGFP-TPX2 concentrations (49, 98, 195, and 390 nM). Assays were performed in the presence of mGFP-TPX2 and 10 μM CF640R-tubulin using 373 pM γTuRC for immobilization. (E) Representative TIRFM images of γTuRC-mediated microtubule nucleation in the presence of mGFP-TPX2 concentrations (green), as indicated. Plots showing, (F) linearly increasing microtubule numbers over time, (G) the mean microtubule nucleation rate, and (H) the mean microtubule growth speed at different mGFP-TPX2 concentrations. Number of microtubule growth speeds measured per conditions: 0 nM, n = 15; 49 nM, n = 50; 98 nM, n = 77; 195 nM, n = 143; 390 nM, n = 105.

(I–L) γTuRC-mediated microtubule nucleation in the presence of different mGFP-EB3 concentrations (50, 100, 200, and 400 nM). Assays were performed in the presence of mGFP-EB3 and 12.5 μM CF640R-tubulin using 373 pM γTuRC for immobilization. (I) Representative merged TIRFM images of γTuRC-mediated microtubule nucleation in the presence of different mGFP-EB3 concentrations (green), as indicated. Plots showing, (J) linearly increasing microtubule numbers over time, (K) the mean microtubule nucleation rate, and (L) the mean microtubule growth speed at different mGFP-EB3 concentrations. Number of microtubule growth speeds measured per conditions: 0 nM, n = 210; 50 nM, n = 132; 100 nM, n = 230; 200 nM, n = 191; 400 nM, n = 290. As controls, representative TIRFM images in either the absence of microtubule associated proteins or absence of γTuRC are shown for the highest tested concentration of the various microtubule-associated proteins. Lines represent the linear regression. Nucleation rates (r_{nuc}) were taken from the slope of the linear regression of the increase of microtubule number over time (see Figures 4B, 4F, and 4J). All experiments were performed at 33°C. All error bars are SEM. For symbols without visible error bars, error bars are smaller than the symbol size. Field of view was always 164 × 164 μm. AU, arbitrary units. Fluorescence intensities are directly comparable. Scale bars as indicated. t = 0 is 2 min after placing the sample at 33°C. See also Figures S3 and S4; Videos S6 and S7.

Table 1. Data Collection and Refinement Statistics

γTuRC Cryo-EM Data Collection and Map Refinement	
Microscope	Titan Krios TEM (Thermo Fisher)
Detector	K2 summit (Gatan) in counting mode
Nominal magnification	130,000
Voltage (kV)	300
Electron exposure ($e^{-}/Å^2$)	50
Defocus range (μm)	−1.0 to −3.5
Pixel size (Å)	1.08
Symmetry	C1
Initial particle images (no.)	1,100,000
Final particle images (no.)	522,496
Sharpening B-factor	−100
FSC threshold	0.143
Map resolution (Å)	4.0
Map resolution range (Å)	3.5 to 8.5

(Figure 6C; see also Data S2). We generated a pseudo-atomic structure by docking homology models into the cryo-EM density and mapped the crosslinks on our structure. The best match between the atomic model and CLMS data contains GCP5 in GCP position 10, sandwiched between two GCP4 molecules in positions 9 and 11 and GCP6 in position 12, sandwiched between GCP4 in position 11, and GCP2 in position 13 (Figure 6C). Only this solution satisfies the physical constraints of the BS3 crosslinker (all inter-GCP crosslinks measure less than 25 Å for positions 9, 10, 11, 12, and 13). Coherent with this solution, GCP5 and GCP6 MID domains display density features that are absent in GCP4, as expected from their unique characteristic MID domain insertions. Furthermore, GCP6 contains a unique N-terminal appendix that can be recognized in GCP position 12 (Figures 6D and 6E). Two crosslinks between the GCP6 and GCP3 core domains visible in our pseudo-atomic models involve amino acids distant more than 30 Å apart. The most likely interpretation is that the GCP2-GCP3 heterodimer in positions 13-14 is highly flexible and could come in closer proximity to GCP6, when engaged in stabilizing interactions, as further addressed in the discussion section.

Peripherally Bound MZT2 and an Internal Actin Appear to Form Stabilizing Contacts

Focusing next on the residual unoccupied density, we noted that specific N-terminal GCP interfaces (in positions 1-2, 3-4, 5-6, 7-8, and 13-14) contacted a discernible Λ-shaped α-helical feature, lining the outer perimeter of the constricted cone end (Figure 7A). This feature appears to seal off the GCP2-GCP3 interface, occupying a position that matches that of Spc110, required for stable γTuSC complex formation in yeast (Kollman et al., 2015) (Figure 7B). CLMS analysis assigns this feature to MZT2, as it crosslinks with GCP2 and GCP3 elements on the outer perimeter of the γTuRC N-terminal constriction. Importantly, 5 of the 6 GCP2-GCP3 residues, which crosslink with MZT2 and are visible in our pseudo-atomic model, are surface exposed and map less than 25 Å away from the Λ-shaped feature (Figure 7B). Conversely MZT1 crosslinks with C-terminal GCP2, N-terminal GCP3, and N-terminal GCP6. No obvious unoccupied features were detected in our cryo-EM map, indicating that MZT1 is only flexibly tethered to the core of the γTuRC assembly. On one end of the spiral, proximal to the γ-tubulin face, we could additionally observe some unassigned C-terminal density contacting the GCP3 Cterminus, which might function as a cap that blocks further GCP polymerization, helping to define subunit composition in the γTuRC complex (Figure 7A).

Additional, prominent density could also be observed within the lumen of the γTuRC cone. Actin co-purifies with γTuRC (Figures 1C, S1B, and S1C) (Choi et al., 2010; Oegema et al., 1999). Attempting to dock one actin protomer (PDB:2HF3 Rould et al., 2006) into the luminal density resulted in an unambiguous fit (cross-correlation coefficient 0.74, against a 0.49 score for a 180° rotated solution) and revealed direct contacts between both γ-tubulin and GCP3 in GCP position 2 (Figure 7C). CLMS analysis confirmed these contacts, for example, a crosslink between GCP3 lysine 276 and actin lysine 113 maps 30 Å apart in our structure, compatible with the linker length in the BS3 crosslinking reagent (Figure 6C). Actin also interacts with one unassigned luminal feature, formed by a helical repeat module, which straddles across the central pore of the helical assembly, bridging between γ-tubulin in position 2 with GCP3 in position 8 (Figure 7D). In our CLMS experiment, GCP3 crosslinked with a GCP6 large N-terminal extension that is missing in our model. Thus, N-terminal GCP6 is a strong candidate for the unassigned luminal feature, coherent with a recently proposed model (Liu et al., 2020) (Figure 6C). No obvious structural change can be detected at our resolution for the actin-engaged γ-tubulin.

In summary, actin and associated luminal factors, as well as MZT2 on the outer perimeter of the assembly appear engaged in stabilizing interactions that hold together the γTuRC spiral. We note that γTuRC protomers in positions 1–8 match the GCP2-GCP3 configuration in the active ("closed") configuration of yeast γTuSC (Kollman et al., 2015). Conversely, subunits in positions 9–14, which notably lack any stabilizing element within the lumen, display a configuration more akin to the inactive ("open") form of yeast γTuSC (Kollman et al., 2015). Coherently, subunits in positions 1–8 appear to match the geometry of a 13-subunit microtubule protofilament and could hence sustain nucleation, possibly starting from position 2 in the γTuRC, which presents the first sterically available docking site on γ-tubulin. The observation that γTuRC subunits in positions 9–14 markedly diverge from microtubule geometry could justify our observation that γTuRC enhanced microtubule nucleation is not very efficient (Video S8).

DISCUSSION

Using TIRF microscopy, we imaged the nucleation of individual microtubules by surface-immobilized human γTuRC. Microtubules were stably capped by γTuRC, not displaying any minus-end dynamics. γTuRC increased the nucleation efficiency compared with microtubule nucleation in solution, but nucleation still had to overcome a significant kinetic barrier. Microtubule formation on the γTuRC template was mechanistically very different from microtubule plus-end elongation. Determining the structure of the human γTuRC complex, using a combination of cryo-EM and CLMS, revealed an asymmetric conformation with half of the complex in a compact configuration (closed) and half containing loosely interacting protomers (open). As observed for budding yeast γTuRC (Kollman et al., 2015), the

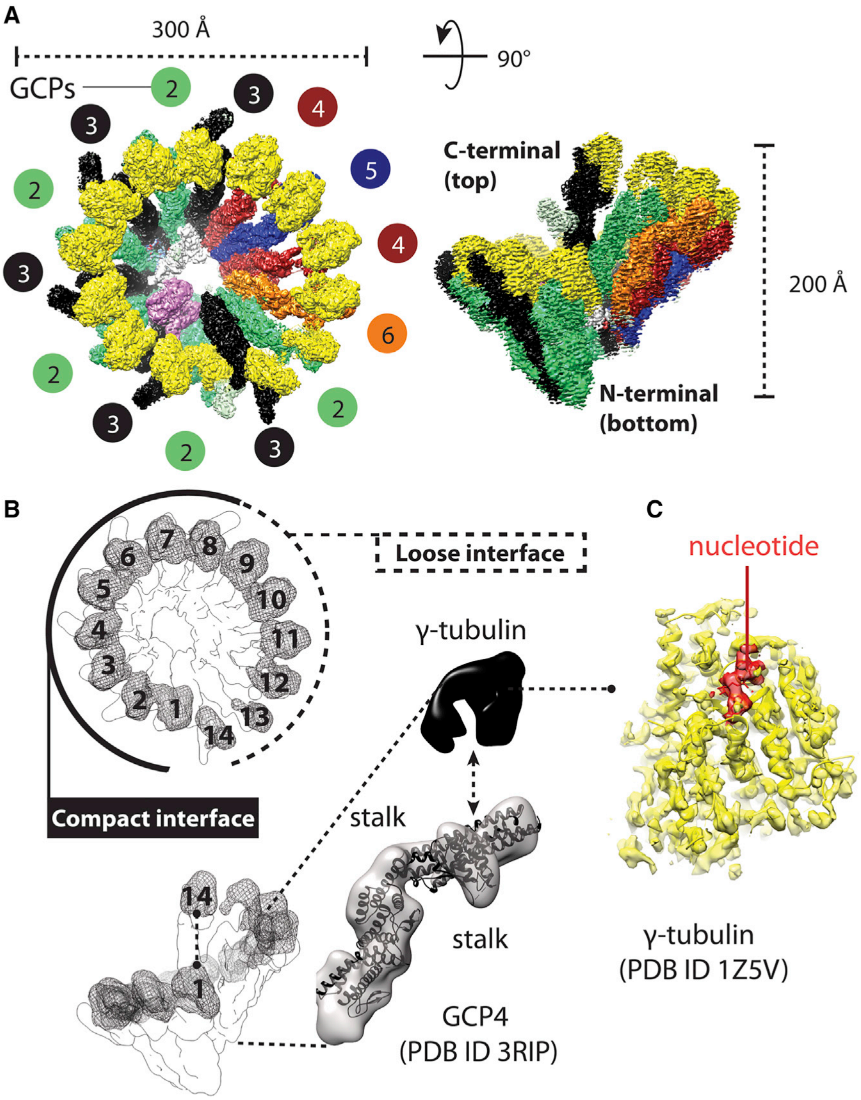

Figure 5. Cryo-EM Structure of Human γTuRC

(A) Surface rendering of the cryo-EM structure viewed from the top and side. γTuRC is shaped like a cone with a base diameter of 300 and height of 200 Å.

(B) γTuRC contains 14 stalk protomers that support 14 globular densities. Subunits in the lowermost position 1 and the uppermost position 14 are aligned. Docking of the human GCP4 crystal structure (PDB:3RIP) into any of the spiral cone positions reveals that the 14 stalk densities correspond to GCP proteins (position 1 is shown here as an example). A low resolution version of the cryo-EM map is shown to focus on the overall shape of the complex.

(C) The 14 globular densities instead correspond to γ-tubulin, as revealed by atomic docking of PDB:1Z5V, here shown docked into position 8 as an example. Nucleotide density (GTPγS in the crystal structure used) is shown in red.

rise of the helical arrangement of the γ-tubulins imposes a "three start helix" microtubule lattice structure with the seam being positioned between γ-tubulin 1 and 2. The human γTuRC structure is however distinctly different from the structure of budding yeast γTuRC (Kollman et al., 2015), particularly where the GCP4, 5, and 6 subunits are located that are absent in yeast γTuRC.

Microtubule nucleation in solution has been described as a highly cooperative process. Many tubulins need to come together to form a first minimal stable assembly (Voter and Erickson, 1984). Although γTuRC is thought to nucleate microtubules by providing a template that mimics the microtubule structure, also nucleation from this template faced a kinetic barrier and was highly cooperative. Our estimate of at least ~7 tubulins needing to come together before stable microtubule outgrowth from the γTuRC complex can occur (Figure 3D) is at the lower end of the reported range of 6–15 tubulins for such a critical nucleus required for spontaneous nucleation in solution (Flyvbjerg and Jobs, 1997; Kuchnir Fygenson et al., 1995; Voter and Erickson, 1984), supporting the notion that the template facilitates nucleation.

However, microtubule outgrowth from the γTuRC template is considerably more difficult than elongation from a pre-existing growing microtubule plus end. Only a fraction of γTuRCs nucleated within 20 min in our assay. We did not observe any indication for a permanently inactive γTuRC population, because the probability of stochastic nucleation was constant over time. However, we cannot exclude that some purified and surface-immobilized complexes are permanently inactive.

Our γTuRC structure can explain the observed inefficient nucleation. Only half of the human γTuRC complex exists in a closed configuration with four GCP2-GCP3 heterodimers bound to 8 γ-tubulins being bridged together by stabilizing factors including one actin monomer in the lumen of the cone-shaped complex. Conversely, the other half of the complex where GCP4-GCP5 and GCP4-GCP6 subunits are located, together with one additional GCP2-GCP3 dimer at the very top of the helical arrangement of GCP dimers, lack bridging luminal elements and only loosely interact with one another. This open configuration causes a γ-tubulin arrangement that deviates from the microtubule geometry and that is expected for an active γTuRC state (Video S8). This asymmetric structure indicates that the purified human γTuRC is not in a fully active conformation. Either microtubule assembly on the γTuRC surface may induce γTuRC closure and/or additional regulatory binding factors may induce a completely closed γTuRC geometry required for efficient nucleation, suggesting a mechanism for the regulation of γTuRC activity.

In favor of the scenario of microtubule assembly-induced γTuRC closure is our observation that proteins, which stabilize growing microtubule ends by different means and which were shown to stimulate microtubule nucleation *in vitro*, also stimulate γTuRC-mediated microtubule nucleation. The microtubule polymerase chTOG may do so by accelerating and thereby

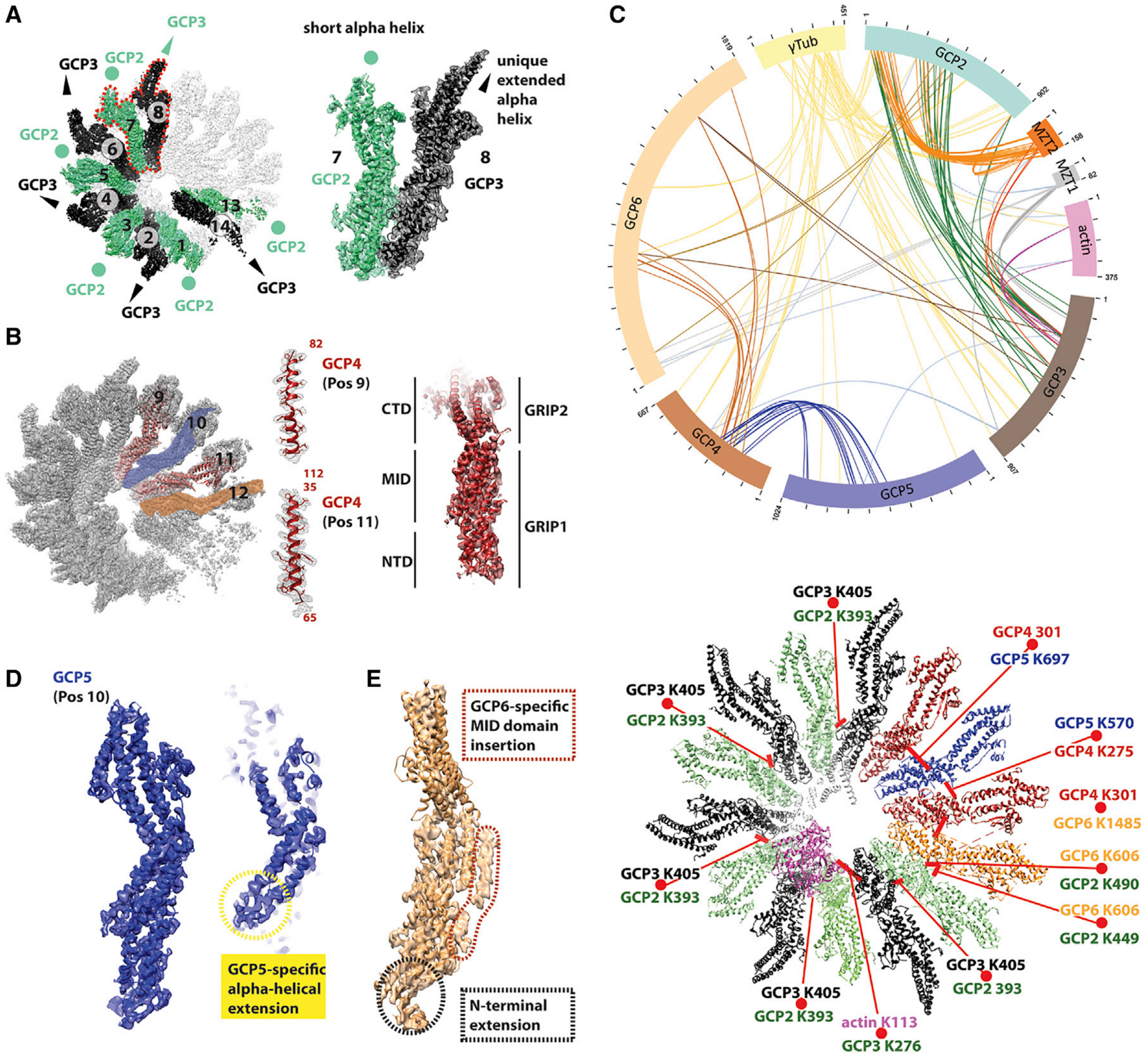

Figure 6. GCP Subunit Assignment

(A) GCP2 and GCP3 are known to form a stable heterodimer. Homology modeling indicates that GCP3 contains a unique α-helical extension, resulting in a distinctive feature that radially departs from the GCP spiral structure. This structural feature allows us to assign GCP2 and GCP3 around the γTuRC complex.
(B) GCP4 can be assign by docking the human crystal structure into the cryo-EM map. This fitting exercise, even in the absence of any further real-space refinement, allows us to appreciate the match between amino acidic side chains from the X-ray model and the density features in the cryo-EM map. We therefore assign GCP4 to positions 9 and 11 in the map. Rigid-body docking of N-terminal (GRIP1) and MID-C-terminal domains (GRIP2) is required to optimize the fitting of each individual structure. Cryo-EM density obtained with Phenix's ResolveCryoEM is shown to highlight the fit of amino acidic side chains (central panel). The cryo-EM density sharpened with RELION post-processing is used elsewhere in the figure.
(C) Top panel: circular representation of the CLMS results. Intra-subunit crosslinks are displayed as purple lines. Inter-subunit crosslinks are represented as green lines. Bottom panel: inter-molecular crosslinks between GCP subunits and actin help establish the subunit order around the γTuRC spiral.
(D) GCP5 in position 10 contains a characteristic predicted helical extension in the MID domain.
(E) Assigned to position 12, GCP6 contains the largest N-terminal extension (marked in black) and MID domain insertion (marked in red) among GCP protomers. Also see Figures S5 and S6.

stabilizing nascent microtubule growth on γTuRC by its polymerase activity (Brouhard et al., 2008; Roostalu et al., 2015), and TPX2 may do so by its catastrophe-suppressing activity (Roostalu et al., 2015; Wieczorek et al., 2015). chTOG and TPX2 were also reported to promote microtubule outgrowth from stabilized microtubule "seeds," suggesting similarities between outgrowth from such seeds and templating a microtubule by γTuRC (Wieczorek et al., 2015). Interestingly, both chTOG and TPX2 have also been reported to directly interact with γTuRC, which may increase the efficiency of their action (Alfaro-Aco et al., 2017; Thawani et al., 2018). However, we did not observe evidence of significant binding of these proteins to our passivated surfaces with specifically immobilized γTuRC. Moreover, TPX2 has been shown to interact additionally with the HAUS (Augmin) complex that promotes branched microtubule nucleation from pre-existing microtubules in mitotic or meiotic spindles during cell division (Alfaro-Aco and Petry, 2017).

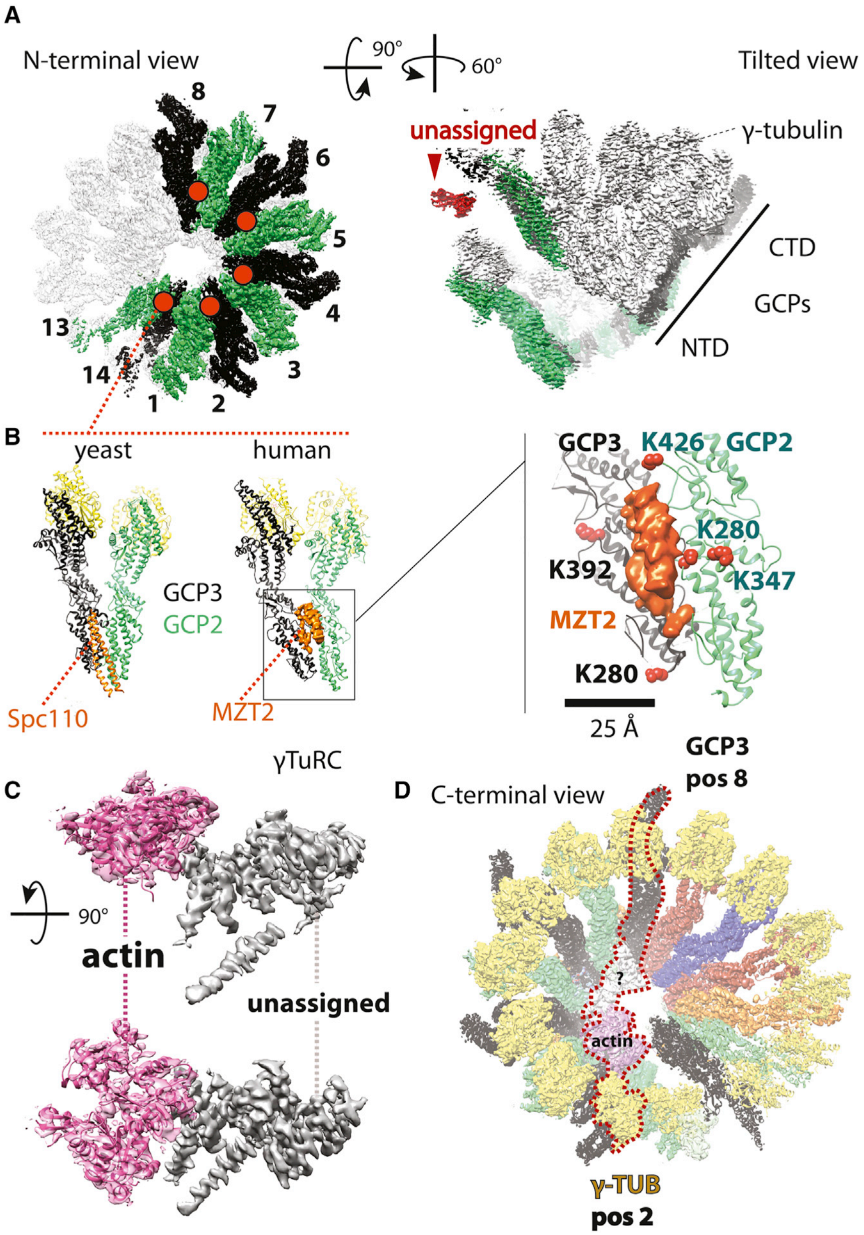

Figure 7. Analysis of the Unassigned Cryo-EM Density in the γTuRC Complex

(A) Unoccupied density appears to seal off the interface of GCP2 and GCP3, lining the outer perimeter of the GCP spiral (marked with an orange circle). A 90° tilted view highlights unassigned density can be observed departing from the C-terminal end of GCP3 in position 14 (shown in red).

(B) The feature on the outer perimeter of the GCP spiral occupies the same position observed for Scp110 in γTuSC. CLMS identifies this feature as MZT2, as this factor is crosslinked with residues clustered on the outer face of the GCP2-GCP3 interface across a region of diameter smaller than 60 Å.

(C) Two orthogonal views corresponding to additional density found in the lumen of the γTuRC spiral. Part of this density can be assigned to actin (magenta), which was found to be co-purified in our preparation. Additional unassigned density shown in gray contains three recognizable alpha helical bundles.

(D) The luminal density bridges γ-tubulin in position 2 and GCP3 in position 8. Also see Figure S6.

Our structure suggests a potential mechanism for the activation of human γTuRC by complex closure. In our structure, all GCP2-GCP3 interfaces in the closed part of the complex are sealed off by MZT2, an inter-protomer element, in a position reminiscent of yeast γTuSC specific Spc110. As for Spc110, we propose that MZT2 is likely required to stabilize the GCP2-GCP3 spiral formation at the constricted N-terminal dimerization core. At least 5 additional binding sites in the open part of the complex remain available at inter-protomer interfaces along the perimeter of the constricted spiral base, which could be engaged by additional nucleation-activation factors.

These elements in the γTuRC structure suggest an activation mechanism for human γTuRC that differs from the activation mechanism for budding yeast γTuRC. This difference is likely a consequence of different GCP subunit compositions of the two complexes. Yeast γTuRC consists of a helical arrangement of 7 identical γTuSCs (2 γ-tubulins and one GCP2 and 3 each) and displays gaps between every second γ-tubulin (within a γTuSC) that create a mismatch with the microtubule geometry (Kollman et al., 2015). Consequently, in addition to regulation at the level of complex assembly by recruitment factors to the spindle pole body, yeast γTuRC can be further activated by a structural change closing the gaps between the γ-tubulins, resulting in a γ-tubulin arrangement that matches the 13-mer protofilament geometry of the microtubule (Kollman et al., 2015). In contrast, the human γTuRC structure departs from the microtubule geometry where GCP4, 5, and 6 are located, resulting in an entire half of the complex being in an open conformation. It is tempting to speculate that other proteins that may line the outer perimeter of the open part of γTuRC (or its inner lumen) may induce a conformational change, closing the second half of the complex and thereby producing a completely closed conformation in which the γ-tubulin configuration closely matches the geometry of the microtubule lattice. Such reconfiguration may consequently reduce the kinetic barrier for templated microtubule nucleation.

Cryo-EM structures of the human and *Xenopus* γTuRC complex have recently been published (Liu et al., 2020; Wieczorek et al., 2020). Overall, these structures are similar to the structure reported here. However, some differences with the previously published structure of the human complex can be observed. An unidentified factor binds between subunits GCP4 and GCP6 in positions 11 and 12, respectively (Wieczorek et al.,

CellPress
OPEN ACCESS

2020), which is absent in our structure of the complex. This factor appears to stabilize the interaction between GCP4 and GCP6, which display a more compact interface than in our structure, possibly representing a partially activated complex.

Moreover, it is interesting to note that the γ-tubulin used in our docking experiment exists in a curved configuration (Aldaz et al., 2005), different from that of tubulin dimers incorporated in a microtubule lattice (Brouhard and Rice, 2014). Additional conformational changes may be required for γ-tubulin as a microtubule assembles on the γTuRC template (Wieczorek et al., 2020).

A major open question for the future will be to understand how the various proteins involved in controlling the efficiency of microtubule nucleation in cells control the conformation and activity of human γTuRC. Real-time *in vitro* nucleation assays in combination with structural investigations will be essential to shed light on the detailed mechanism of spatio-temporal γTuRC activation, probably by an open-to-closed transition.

STAR★METHODS

Detailed methods are provided in the online version of this paper and include the following:

- KEY RESOURCES TABLE
- RESOURCE AVAILABILITY
  - Lead Contact
  - Materials Availability
  - Data and Code Availability
- EXPERIMENTAL MODEL AND SUBJECT DETAILS
- METHOD DETAILS
  - Lentivirus Expression Constructs and Molecular Biology
  - Antibodies
  - Cell Culture and Cell Line Development
  - Purification of Human γTuRC
  - Purification of Human chTOG-mGFP, mGFP-TPX2 and mGFP-EB3
  - Tubulin Purification and Labelling
  - LC-MS/MS Analysis of Fluorescently Tagged γTuRC
  - γTuRC-Mediated Microtubule Nucleation Assay
  - Microtubule Dynamics Assays Using 'Seeds'
  - TIRF Microscopy
  - TIRF Microscopy Image Processing
  - Microtubule Growth Speeds
  - Microtubule Nucleation Rate Analysis
  - Negative Stain Grid Preparation and Data Collection
  - Cryo Grid Preparation and Data Collection
  - Negative Stain Electron Microscopy Image Processing
  - Cryo-EM Image Processing
  - Generation of an Atomic Model
  - Crosslinking and Mass Spectrometry
- QUANTIFICATION AND STATISTICAL ANALYSIS

SUPPLEMENTAL INFORMATION

Supplemental Information can be found online at https://doi.org/10.1016/j.devcel.2020.04.019.

ACKNOWLEDGMENTS

We thank Andrea Nans at the Structural Biology Science Technology Platform (STP) for support on the Titan Krios, Raffa Carzaniga at the Electron Microscopy STP of the Francis Crick Institute for support on the Tecnai G2 Spirit electron microscope, Nicholas I. Cade for fluorescence microscopy support, and Andrew Purkiss and Phil Walker for computational support. We thank the Cell Services, the flow cytometry, and the mass spectrometry proteomics STPs of the Francis Crick Institute for producing large cell cultures and for cell sorting and protein identification support. This work was supported by the Francis Crick Institute, which receives its core funding from Cancer Research UK (FC001163 and FC0010065), the UK Medical Research Council (FC001163 and FC0010065), and the Wellcome Trust (FC001163 and FC0010065) to T.S. and A.C. The Wellcome Centre for Cell Biology is supported by core funding from the Wellcome Trust (203149). J. Rappsilber is funded by the Deutsche Forschungsgemeinschaft (DFG, German Research Foundation) under Germany's Excellence Strategy – EXC 2008 – 390540038 – UniSysCat and 329673113. J. Roostalu. was supported by a Sir Henry Wellcome Postdoctoral Fellowship (100145/Z/12/Z) and M.A.C. is supported by a Marie Skłodowska-Curie Postdoctoral Fellowship (agreement no. 845939). T.S. acknowledges support from the European Research Council (Advanced Grant, project 323042). A.C. receives funding from the European Research Council (ERC) under the European Union's Horizon 2020 research and innovation program (grant agreement no. 820102). T.C., J.A., J.W.M., and T.S. acknowledge also the support of the Spanish Ministry of Economy, Industry and Competitiveness to the CRG-EMBL partnership, the Centro de Excelencia Severo Ochoa and the CERCA Programme of the Generalitat de Catalunya.

AUTHOR CONTRIBUTIONS

Conceptualization, T.C., J.L., J. Roostalu, A.C., and T.S.; Methodology, T.C., J.L., J. Roostalu, Z.A.C., J.G., J.A., W.M.L., and M.A.C; Validation, T.C. and J.L.; Formal Analysis, T.C., J.L., Z.A.C., F.M., J. Rappsilber, and A.C.; Investigation, T.C., J.L., Z.A.C., J.G., J.A., W.M.L., and M.A.C.; Resources, T.C., J. Roostalu, J.G., J.A., and W.M.L., Original Draft, T.C., A.C., and T.S.; Review & Editing, T.C., J.L., Z.A.C., J.A., W.M.L., J. Rappsilber, A.C., and T.S.; Visualization, T.C., J.L., Z.A.C., F.M., J. Rappsilber, and A.C.; Supervision, J. Roostalu, J. Rappsilber, A.C., and T.S.; Project Administration, T.C., J.L., A.C., and T.S.; Funding Acquisition, M.A.C., J. Rappsilber, A.C., and T.S.

DECLARATION OF INTERESTS

The authors declare no competing interests.

Received: December 2, 2019
Revised: March 21, 2020
Accepted: April 27, 2020
Published: May 19, 2020

REFERENCES

Abella, J.V., Galloni, C., Pernier, J., Barry, D.J., Kjær, S., Carlier, M.F., and Way, M. (2016). Isoform diversity in the Arp2/3 complex determines actin filament dynamics. Nat. Cell Biol. *18*, 76–86.

Adams, P.D., Afonine, P.V., Bunkóczi, G., Chen, V.B., Davis, I.W., Echols, N., Headd, J.J., Hung, L.W., Kapral, G.J., Grosse-Kunstleve, R.W., et al. (2010). PHENIX: a comprehensive Python-based system for macromolecular structure solution. Acta Crystallogr. D Biol. Crystallogr. *66*, 213–221.

Afonine, P.V., Poon, B.K., Read, R.J., Sobolev, O.V., Terwilliger, T.C., Urzhumtsev, A., and Adams, P.D. (2018). Real-space refinement in PHENIX for cryo-EM and crystallography. Acta Crystallogr D Struct Biol *74*, 531–544.

Akhmanova, A., and Steinmetz, M.O. (2015). Control of microtubule organization and dynamics: two ends in the limelight. Nat. Rev. Mol. Cell Biol. *16*, 711–726.

Aldaz, H., Rice, L.M., Stearns, T., and Agard, D.A. (2005). Insights into microtubule nucleation from the crystal structure of human gamma-tubulin. Nature *435*, 523–527.

Alfaro-Aco, R., and Petry, S. (2017). How TPX2 helps microtubules branch out. Cell Cycle *16*, 1560–1561.

Alfaro-Aco, R., Thawani, A., and Petry, S. (2017). Structural analysis of the role of TPX2 in branching microtubule nucleation. J. Cell Biol. *216*, 983–997.

Bieling, P., Telley, I.A., Hentrich, C., Piehler, J., and Surrey, T. (2010). Fluorescence microscopy assays on chemically functionalized surfaces for quantitative imaging of microtubule, motor, and +TIP dynamics. Methods Cell Biol. *95*, 555–580.

Brouhard, G.J., and Rice, L.M. (2014). The contribution of αβ-tubulin curvature to microtubule dynamics. J. Cell Biol. *207*, 323–334.

Brouhard, G.J., and Rice, L.M. (2018). Microtubule dynamics: an interplay of biochemistry and mechanics. Nat. Rev. Mol. Cell Biol. *19*, 451–463.

Brouhard, G.J., Stear, J.H., Noetzel, T.L., Al-Bassam, J., Kinoshita, K., Harrison, S.C., Howard, J., and Hyman, A.A. (2008). XMAP215 is a processive microtubule polymerase. Cell *132*, 79–88.

Castoldi, M., and Popov, A.V. (2003). Purification of brain tubulin through two cycles of polymerization-depolymerization in a high-molarity buffer. Protein Expr. Purif. *32*, 83–88.

Choi, Y.K., Liu, P., Sze, S.K., Dai, C., and Qi, R.Z. (2010). CDK5RAP2 stimulates microtubule nucleation by the gamma-tubulin ring complex. J. Cell Biol. *191*, 1089–1095.

Cota, R.R., Teixidó-Travesa, N., Ezquerra, A., Eibes, S., Lacasa, C., Roig, J., and Lüders, J. (2017). MZT1 regulates microtubule nucleation by linking γTuRC assembly to adapter-mediated targeting and activation. J. Cell Sci. *130*, 406–419.

Emsley, P., Lohkamp, B., Scott, W.G., and Cowtan, K. (2010). Features and development of coot. Acta Crystallogr. D Biol. Crystallogr. *66*, 486–501.

Erickson, H.P., and Pantaloni, D. (1981). The role of subunit entropy in cooperative assembly. Nucleation of microtubules and other two-dimensional polymers. Biophys. J. *34*, 293–309.

Farache, D., Emorine, L., Haren, L., and Merdes, A. (2018). Assembly and regulation of gamma-tubulin complexes. Open Biol. *8*, 170266.

Fischer, L., and Rappsilber, J. (2017). Quirks of error estimation in cross-linking/mass spectrometry. Anal. Chem. *89*, 3829–3833.

Flyvbjerg, H., and Jobs, E. (1997). Microtubule dynamics.II. Kinetics of self-assembly. Phys. Rev. E *56*, 7083–7099.

Gard, D.L., and Kirschner, M.W. (1987a). Microtubule assembly in cytoplasmic extracts of Xenopus oocytes and eggs. J. Cell Biol. *105*, 2191–2201.

Gard, D.L., and Kirschner, M.W. (1987b). A microtubule-associated protein from Xenopus eggs that specifically promotes assembly at the plus-end. J. Cell Biol. *105*, 2203–2215.

Ghosh, S., Hentrich, C., and Surrey, T. (2013). Micropattern-controlled local microtubule nucleation, transport, and mesoscale organization. ACS Chem. Biol. *8*, 673–678.

Gruss, O.J., Carazo-Salas, R.E., Schatz, C.A., Guarguaglini, G., Kast, J., Wilm, M., Le Bot, N., Vernos, I., Karsenti, E., and Mattaj, I.W. (2001). Ran induces spindle assembly by reversing the inhibitory effect of importin alpha on TPX2 activity. Cell *104*, 83–93.

Guillet, V., Knibiehler, M., Gregory-Pauron, L., Remy, M.H., Chemin, C., Raynaud-Messina, B., Bon, C., Kollman, J.M., Agard, D.A., Merdes, A., and Mourey, L. (2011). Crystal structure of gamma-tubulin complex protein GCP4 provides insight into microtubule nucleation. Nat. Struct. Mol. Biol. *18*, 915–919.

Gunawardane, R.N., Martin, O.C., Cao, K., Zhang, L., Dej, K., Iwamatsu, A., and Zheng, Y. (2000). Characterization and reconstitution of Drosophila gamma-tubulin ring complex subunits. J. Cell Biol. *151*, 1513–1524.

Gunzelmann, J., Rüthnick, D., Lin, T.C., Zhang, W., Neuner, A., Jäkle, U., and Schiebel, E. (2018). The microtubule polymerase Stu2 promotes oligomerization of the gamma-TuSC for cytoplasmic microtubule nucleation. eLife *7*, e39932.

Hannabuss, J., Lera-Ramirez, M., Cade, N.I., Fourniol, F.J., Nédélec, F., and Surrey, T. (2019). Self-organization of minimal anaphase spindle midzone bundles. Curr. Biol. *29*, 2120–2130.e7.

Hutchins, J.R., Toyoda, Y., Hegemann, B., Poser, I., Hériché, J.K., Sykora, M.M., Augsburg, M., Hudecz, O., Buschhorn, B.A., Bulkescher, J., et al. (2010). Systematic analysis of human protein complexes identifies chromosome segregation proteins. Science *328*, 593–599.

Hyman, A., Drechsel, D., Kellogg, D., Salser, S., Sawin, K., Steffen, P., Wordeman, L., and Mitchison, T. (1991). Preparation of modified tubulins. Methods Enzymol. *196*, 478–485.

Jha, R., Roostalu, J., Cade, N.I., Trokter, M., and Surrey, T. (2017). Combinatorial regulation of the balance between dynein microtubule end accumulation and initiation of directed motility. EMBO J *36*, 3387–3404.

Kidmose, R.T., Juhl, J., Nissen, P., Boesen, T., Karlsen, J.L., and Pedersen, B.P. (2019). Namdinator - automatic molecular dynamics flexible fitting of structural models into cryo-EM and crystallography experimental maps. IUCrJ *6*, 526–531.

King, M.R., and Petry, S. (2020). Phase separation of TPX2 enhances and spatially coordinates microtubule nucleation. Nat. Commun. *11*, 270.

Kollman, J.M., Greenberg, C.H., Li, S., Moritz, M., Zelter, A., Fong, K.K., Fernandez, J.J., Sali, A., Kilmartin, J., Davis, T.N., and Agard, D.A. (2015). Ring closure activates yeast γTuRC for species-specific microtubule nucleation. Nat. Struct. Mol. Biol. *22*, 132–137.

Kollman, J.M., Merdes, A., Mourey, L., and Agard, D.A. (2011). Microtubule nucleation by gamma-tubulin complexes. Nat. Rev. Mol. Cell Biol. *12*, 709–721.

Kollman, J.M., Polka, J.K., Zelter, A., Davis, T.N., and Agard, D.A. (2010). Microtubule nucleating gamma-TuSC assembles structures with 13-fold microtubule-like symmetry. Nature *466*, 879–882.

Kronja, I., Kruljac-Letunic, A., Caudron-Herger, M., Bieling, P., and Karsenti, E. (2009). XMAP215-EB1 interaction is required for proper spindle assembly and chromosome segregation in Xenopus egg extract. Mol. Biol. Cell *20*, 2684–2696.

Kuchnir Fygenson, D.K., Flyvbjerg, H., Sneppen, K., Libchaber, A., and Leibler, S. (1995). Spontaneous nucleation of microtubules. Phys. Rev. E *51*, 5058–5063.

Lin, T.C., Neuner, A., Flemming, D., Liu, P., Chinen, T., Jäkle, U., Arkowitz, R., and Schiebel, E. (2016). MOZART1 and gamma-tubulin complex receptors are both required to turn gamma-TuSC into an active microtubule nucleation template. J. Cell Biol. *215*, 823–840.

Lin, T.C., Neuner, A., and Schiebel, E. (2015). Targeting of gamma-tubulin complexes to microtubule organizing centers: conservation and divergence. Trends Cell Biol. *25*, 296–307.

Lin, T.C., Neuner, A., Schlosser, Y.T., Scharf, A.N., Weber, L., and Schiebel, E. (2014). Cell-cycle dependent phosphorylation of yeast pericentrin regulates gamma-TuSC-mediated microtubule nucleation. eLife *3*, e02208.

Liu, P., Zupa, E., Neuner, A., Böhler, A., Loerke, J., Flemming, D., Ruppert, T., Rudack, T., Peter, C., Spahn, C., et al. (2020). Insights into the assembly and activation of the microtubule nucleator gamma-TuRC. Nature *578*, 467–471.

Maurer, S.P., Cade, N.I., Bohner, G., Gustafsson, N., Boutant, E., and Surrey, T. (2014). EB1 accelerates two conformational transitions important for microtubule maturation and dynamics. Curr. Biol. *24*, 372–384.

Mendes, M.L., Fischer, L., Chen, Z.A., Barbon, M., O'Reilly, F.J., Giese, S.H., Bohlke-Schneider, M., Belsom, A., Dau, T., Combe, C.W., et al. (2019). An integrated workflow for crosslinking mass spectrometry. Mol. Syst. Biol. *15*, e8994.

Muroyama, A., Seldin, L., and Lechler, T. (2016). Divergent regulation of functionally distinct gamma-tubulin complexes during differentiation. J. Cell Biol. *213*, 679–692.

Murphy, S.M., Preble, A.M., Patel, U.K., O'Connell, K.L., Dias, D.P., Moritz, M., Agard, D., Stults, J.T., and Stearns, T. (2001). GCP5 and GCP6: two new members of the human gamma-tubulin complex. Mol. Biol. Cell *12*, 3340–3352.

Oegema, K., Wiese, C., Martin, O.C., Milligan, R.A., Iwamatsu, A., Mitchison, T.J., and Zheng, Y. (1999). Characterization of two related Drosophila gamma-tubulin complexes that differ in their ability to nucleate microtubules. J. Cell Biol. *144*, 721–733.

Pettersen, E.F., Goddard, T.D., Huang, C.C., Couch, G.S., Greenblatt, D.M., Meng, E.C., and Ferrin, T.E. (2004). UCSF chimera–a visualization system for exploratory research and analysis. J. Comput. Chem. *25*, 1605–1612.

Popov, A.V., Severin, F., and Karsenti, E. (2002). XMAP215 is required for the microtubule-nucleating activity of centrosomes. Curr. Biol. *12*, 1326–1330.

Punjani, A., Rubinstein, J.L., Fleet, D.J., and Brubaker, M.A. (2017). cryoSPARC: algorithms for rapid unsupervised cryo-EM structure determination. Nat. Methods *14*, 290–296.

Reber, S.B., Baumgart, J., Widlund, P.O., Pozniakovsky, A., Howard, J., Hyman, A.A., and Jülicher, F. (2013). XMAP215 activity sets spindle length by controlling the total mass of spindle microtubules. Nat. Cell Biol. *15*, 1116–1122.

Roostalu, J., Cade, N.I., and Surrey, T. (2015). Complementary activities of TPX2 and chTOG constitute an efficient importin-regulated microtubule nucleation module. Nat. Cell Biol. *17*, 1422–1434.

Roostalu, J., and Surrey, T. (2017). Microtubule nucleation: beyond the template. Nat. Rev. Mol. Cell Biol. *18*, 702–710.

Roostalu, J., Thomas, C., Cade, N.I., Kunzelmann, S., Taylor, I.A., and Surrey, T. (2020). The speed of GTP hydrolysis determines GTP cap size and controls microtubule stability. eLife *9*, e51992.

Rould, M.A., Wan, Q., Joel, P.B., Lowey, S., and Trybus, K.M. (2006). Crystal structures of expressed non-polymerizable monomeric actin in the ADP and ATP states. J. Biol. Chem. *281*, 31909–31919.

Roy, A., Kucukural, A., and Zhang, Y. (2010). I-TASSER: a unified platform for automated protein structure and function prediction. Nat. Protoc. *5*, 725–738.

Schatz, C.A., Santarella, R., Hoenger, A., Karsenti, E., Mattaj, I.W., Gruss, O.J., and Carazo-Salas, R.E. (2003). Importin alpha-regulated nucleation of microtubules by TPX2. EMBO J. *22*, 2060–2070.

Scheres, S.H. (2012). RELION: implementation of a Bayesian approach to cryo-EM structure determination. J. Struct. Biol. *180*, 519–530.

Scrofani, J., Sardon, T., Meunier, S., and Vernos, I. (2015). Microtubule nucleation in mitosis by a RanGTP-dependent protein complex. Curr. Biol. *25*, 131–140.

Tang, G., Peng, L., Baldwin, P.R., Mann, D.S., Jiang, W., Rees, I., and Ludtke, S.J. (2007). EMAN2: an extensible image processing suite for electron microscopy. J. Struct. Biol. *157*, 38–46.

Teixidó-Travesa, N., Roig, J., and Lüders, J. (2012). The where, when and how of microtubule nucleation - one ring to rule them all. J. Cell Sci. *125*, 4445–4456.

Terwilliger, T.C., Ludtke, S.J., Read, R.J., Adams, I.R., and Afonine, P.V. (2019). Improvement of cryo-EM maps by density modification. bioRxiv. https://doi.org/10.1101/845032v1.

Thawani, A., Kadzik, R.S., and Petry, S. (2018). XMAP215 is a microtubule nucleation factor that functions synergistically with the gamma-tubulin ring complex. Nat. Cell Biol. *20*, 575–585.

Thawani, A., Stone, H.A., Shaevitz, J.W., and Petry, S. (2019). Spatiotemporal organization of branched microtubule networks. eLife *8*, e43890.

Tovey, C.A., and Conduit, P.T. (2018). Microtubule nucleation by gamma-tubulin complexes and beyond. Essays Biochem. *62*, 765–780.

Voter, W.A., and Erickson, H.P. (1984). The kinetics of microtubule assembly. Evidence for a two-stage nucleation mechanism. J. Biol. Chem. *259*, 10430–10438.

Wagner, T., Merino, F., Stabrin, M., Moriya, T., Antoni, C., Apelbaum, A., Hagel, P., Sitsel, O., Raisch, T., Prumbaum, D., et al. (2019). SPHIRE-crYOLO is a fast and accurate fully automated particle picker for cryo-EM. Commun. Biol. *2*, 218.

Wieczorek, M., Bechstedt, S., Chaaban, S., and Brouhard, G.J. (2015). Microtubule-associated proteins control the kinetics of microtubule nucleation. Nat. Cell Biol. *17*, 907–916.

Wieczorek, M., Urnavicius, L., Ti, S.C., Molloy, K.R., Chait, B.T., and Kapoor, T.M. (2020). Asymmetric Molecular Architecture of the Human gamma-tubulin Ring Complex. Cell *180*, 165–175.e16.

Wühr, M., Freeman, R.M., Jr., Presler, M., Horb, M.E., Peshkin, L., Gygi, S., and Kirschner, M.W. (2014). Deep proteomics of the Xenopus laevis egg using an mRNA-derived reference database. Curr. Biol. *24*, 1467–1475.

Zhang, K. (2016). Gctf: real-time CTF determination and correction. J. Struct. Biol. *193*, 1–12.

Zheng, S.Q., Palovcak, E., Armache, J.P., Verba, K.A., Cheng, Y., and Agard, D.A. (2017). MotionCor2: anisotropic correction of beam-induced motion for improved cryo-electron microscopy. Nat. Methods *14*, 331–332.

Zheng, Y., Wong, M.L., Alberts, B., and Mitchison, T. (1995). Nucleation of microtubule assembly by a gamma-tubulin-containing ring complex. Nature *378*, 578–583.

Zivanov, J., Nakane, T., Forsberg, B.O., Kimanius, D., Hagen, W.J., Lindahl, E., and Scheres, S.H. (2018). New tools for automated high-resolution cryo-EM structure determination in RELION-3. eLife *7*, e42166.

STAR★METHODS

KEY RESOURCES TABLE

REAGENT or RESOURCE	SOURCE	IDENTIFIER
Antibodies		
γ-tubulin, clone GTU-88	Sigma-Aldrich	Cat#: T6557; RRID: AB_477584
mTagBFP	Evrogen	Cat#: AB233; RRID: AB_2571743
polyclonal rabbit anti-GCP2 antibody (amino acids 1-155)	this study	costum-made, Pettingill
polyclonal rabbit anti-GCP4 antibody (amino acids: 1-745)	this study	costum-made Covalab
mouse anti-HA, clone F-7	Santa Cruz Biotechnology	Cat#: sc-7392; RRID: AB_627809
anti-rabbit WestVision Peroxidase Polymer antibody	Vector	Cat#: WB-1000; RRID: AB_2336860
goat anti-mouse immunoglobulins/HRP	Agilent	Cat#: P0447; RRID: AB_2617137
goat anti-mouse (H+L) antibody, FITC conjugate	Sigma-Aldrich	Cat#: 12-506; RRID: AB_390186
rabbit anti-actin, beta polyclonal antibody	Abcam	Cat# ab8227; RRID:AB_2305186
Bacterial and Virus Strains		
Bacterial strain for molecular cloning: *Escherichia coli* DH5α	EMBL	Strain name: DH5α
Chemicals, Peptides, and Recombinant Proteins		
γTuRC-GCP2-mBFP-AviTag	This study	Corresponding recombinant DNA: pTC069
mGFP-EB3	Previously used by Roostalu et al. (2020)	N/A
mGFP-TPX2	Previously used by Roostalu et al. (2015)	N/A
chTOG-mGFP	Previously used by Roostalu et al. (2015)	N/A
Pig brain tubulin	Purified according to Castoldi and Popov (2003)	N/A
Catalase	Sigma-Aldrich	Cat#: C40
Glucose Oxidase	Serva	Cat#: 22778.01
Bovine Serum Albumin	Sigma-Aldrich	Cat#: 05470
κ-casein	Sigma-Aldrich	Cat#: C0406
NeutrAvidin	LifeTechnologies	Cat#: A2666
(3-Glycidyloxypropyl)trimethoxy-silane	Sigma-Aldrich	Cat#: 440167
Biotin-CONH-PEG-NH_2 (3000 Da)	Rapp Polymere GmbH	Cat#: 133000-25-20
HO-PEG-NH_2 (3000 Da)	Rapp Polymere GmbH	Cat#: 103000-20
Streptavidin-HRP	Thermo Fisher	Cat#: 21130
BS3 (bis(sulfosuccinimidyl)suberate)	Thermo Fisher	Cat#: 21586
Deposited Data		
Human γTuRC	This study	EMD-10744
Crosslinking mass spectrometry data	This study	PRIDE-PXD018106
Experimental Models: Cell Lines		
HeLa Kyoto cells for recombinant GCP2-mBFP-AviTag expression	Cell services, Francis Crick Institute	CVCL_1922

(*Continued on next page*)

Continued

REAGENT or RESOURCE	SOURCE	IDENTIFIER
**Oligonucleotides**		
Primers for GCP2-mBFP-AviTag in pLVX-Puro: GGACTCAGATCT CGAATGAGTGAATTTCGGATTC ACCAT, TGATCAGTTCTTCGCT TCCGCCTCCTCCGCCCTCGTG CCACTCGATCTTCTGAGCCTCG AAGATGTCGTTCAGACCGCCCT GAAAATACAGGTTTTCTCCGCC TCCTCCGCCCTGTGCGGTGAC TGCGACC, AGCGAAGAACTGA TCAAAGAAAAC, GGTAGAATTA TCTAGTCAGTTCAGTTTATGAC CCAGTTT	Sigma-Aldrich	N/A
Primers for HA-BirA in pLVX-IRES-Hyg: CGGTGAATTCCT CGAATGTACCCATACGATG TTCCAGATTACGCTGGCGG AGGAGGCGGAAAGGATAAC ACCGTGCCACTG, AGAGGG GCGGGATCTTATTATTTTTCT GCACTACGCAGG	Sigma-Aldrich	N/A
**Recombinant DNA**		
pTC069 (pLVX-Puro-GCP2-mGFP-AviTag)	This study	cDNA from Origene (NCBI Reference Sequence: NM_001256617.1)
pTC070 (pLVX-IRES-Hyg-HA-BirA)	This study	BirA sequence taken from plasmid pJR284
**Software and Algorithms**		
Fiji for image analysis	NIH, USA	https://fiji.sc/
Matlab for image alignment	MathWorks	https://www.mathworks.com/products/matlab.html
RELION-3.0	Scheres, 2012; Zivanov et al., 2018	https://www2.mrc-lmb.cam.ac.uk/relion/index.php?title=Main_Page
EMAN2 v2.07	Tang et al., 2007	https://blake.bcm.edu/emanwiki/EMAN2
Gctf v.1.18	Zhang, 2016	https://doi.org/10.1016/j.jsb.2015.11.003
MotionCor2	Zheng et al., 2017	https://msg.ucsf.edu/em/software/motioncor2.html
crYOLO (SPHIRE Package)	Wagner et al., 2019	https://msg.ucsf.edu/em/software/motioncor2.html
cryoSPARC v2	Punjani et al., 2017	https://www.nature.com/articles/nmeth.4169
PHENIX v1.13	Adams et al., 2010; Afonine et al., 2018; Terwilliger et al., 2019	http://www.phenix-online.org/
UCSF Chimera	Pettersen et al., 2004	https://www.cgl.ucsf.edu/chimera/
iTasser	Roy et al., 2010	https://www.nature.com/articles/nprot.2010.5
Coot v0.8.8	Emsley et al., 2010	http://scripts.iucr.org/cgi-bin/paper?S0907444910007493
Namdinator	Kidmose et al., 2019	http://journals.iucr.org/m/issues/2019/04/00/eh5002/index.html
xiSEARCH	Mendes et al., 2019	https://www.rappsilberlab.org/software/xisearch
xiFDR	Fischer and Rappsilber, 2017	https://www.rappsilberlab.org/software/xifdr

(*Continued on next page*)

Continued

REAGENT or RESOURCE	SOURCE	IDENTIFIER
Other		
HiPrep 26/10 Desalting column	GE Healthcare	Cat#: 17508701
HiTrap Desalting column	GE Healthcare	Cat#: 17140801
HiTrap SP Sepharose FF column	GE Healthcare	Cat#: 17505401
Streptavidin mutein matrix	Sigma-Aldrich	Cat#: 3708152001
Superose 6 10/300 GL column	GE Healthcare	Cat#: 29091596
Superdex Peptide 3.2/300 column	GE Healthcare	N/A
Lacey grids (400 mesh) with a layer of ultra-thin carbon	Agar Scientific	Cat#: AGS187-4
50-centimetre EASY-Spray C18 LC column	Thermo Scientific	N/A

RESOURCE AVAILABILITY

Lead Contact

Further information and requests for resources and reagents should be directed to and will be fulfilled by the Lead Contact Thomas Surrey (thomas.surrey@crg.eu).

Materials Availability

Plasmids and the cell line generated in this study are available upon request.

Data and Code Availability

The electron microscopy map has been deposited to the Electron Microscopy Data Bank under accession numbers EMD-10744. The mass spectrometry proteomics data have been deposited to the ProteomeXchange Consortium via the PRIDE partner repository with the dataset identifier PXD018106.

EXPERIMENTAL MODEL AND SUBJECT DETAILS

Escherichia coli bacterial strains DH5a and DH10MultiBac were grown in Luria Bertani (LB) medium in the appropriate antibiotics. HeLa-Kyoto cells (RRID:CVCL_1922) were cultured at 37°C (10% CO_2) in Dulbecco's Modified Eagle Medium (DMEM) supplemented with 10% fetal bovine serum, 50 U mL^{-1} penicillin and 50 μg mL^{-1} streptomycin. Absence of mycoplasma contamination was verified regularly.

METHOD DETAILS

Lentivirus Expression Constructs and Molecular Biology

To generate a fluorescently-tagged and biotinylatable human γTuRC, the coding region for full-length human GCP2 (amino acids 1-902) was amplified by PCR using its cDNA as template (NM_001256617.1, Origene). The mTagBFP (blue fluorescent protein, Evrogen) coding sequence was also amplified by PCR. Both PCR-amplified sequences were cloned into a pLVX-Puro vector (Clonetech) using Gibson assembly (In-Fusion cloning, Takara), to form GCP2_G_5A_TEV_G_5A_mTagBFP_G_5A_BAP, an expression construct for GCP2 which is C-terminally tagged with mTagBFP and biotin acceptor peptide (BAP: GLNDIFEAQKIEWHE), both separated from GCP2 by a TEV protease cleavage site. Glycine linkers (G_5A) were placed between sequences. To facilitate the *in vivo* biotinylation of tagged γTuRC *E. coli* biotin ligase BirA was cloned into a pLVX-IRES-Hyg vector (Clonetech) using Gibson assembly to form HA_G_5A_BirA; an expression construct of BirA with an HA-tag added to the BirA N-terminus, separated by a G_5A-linker. Primers used for cloning are listed in the Key Resources Table.

Antibodies

Commercial and custom-made antibodies were used for the characterization of purified γTuRC by western blotting (see Key Resources Table). Custom-made antibodies were raised against His_6-tagged proteins expressed and purified from *E. coli*. Specific antibodies were affinity purified by standard methods using MBP-tagged proteins expressed and purified from *E. coli* and coupled to CNBr-beads (GE Healthcare). The specificity of custom-made antibodies was confirmed by western blotting against human cell lysate after RNAi depletion of target proteins for 72 h using the RNAiMAX Transfection procedure (Thermo Fisher) and the RNA oligonucleotide sequences described previously (Cota et al., 2017). For detection of biotinylated proteins by western blot, peroxidase coupled streptavidin (streptavidin-HRP, Thermo Fisher) was used.

Cell Culture and Cell Line Development

To generate HeLa-Kyoto cells stably expressing biotinylated mTagBFP-tagged GCP2, cells were co-transduced with GCP2 and BirA lentivirus (Abella et al., 2016) followed by hygromycin and puromycin selection. Resistant cells expressing mTagBFP were sorted by FACS (fluorescent assisted cell sorter) and cultured independently in 96 well plates. The isolated single-cell colonies were screened for HA-BirA expression by immunofluorescence staining (primary antibody: mouse anti-HA (F-7, Santa Cruz Biotechnology); secondary antibody: goat anti-mouse-FITC (Sigma)) and then using high throughput imaging (High throughput screening facility, Francis Crick Institute). The localisation of GCP2-mTagBFP-BAP was confirmed by live-cell fluorescence imaging using a spinning disc confocal microscope based on a NikonTI-E frame with a 100x 1.49 N.A. Nikon objective lens (Cairn Research, Faversham, UK). mTagBFP expressing colonies were further tested by western blotting to confirm the expression of GCP2-mTagBFP-BAP and HA-BirA.

When producing large cell cultures for purification, three days before harvesting cells (using trypsination), D-biotin (Sigma Aldrich) was added to a final concentration of 50 μM. Cell pellets were stored at -80°C until further use.

Purification of Human γTuRC

Cells were resuspended in lysis buffer (50 mM HEPES, 150 mM KCl, 5 mM $MgCl_2$, 1 mM EGTA, 1 mM DTT, 0.1 mM GTP, pH 7.4) containing protease inhibitors (complete EDTA-free protease inhibitor mix, Roche) and DNAse I (10 μg ml^{-1}, Sigma-Aldrich). Resuspended cells were lysed using a polytron tissue dispenser (3x90 s at $6.6x10^3$ rpm) and lysate was clarified twice by centrifugation (17,000xg, 15 min, 4°C). Clarified lysate was filtered through three sets of filters with decreasing pore size: 1.2 μm (GE Healthcare), 0.8 μm (GE-Healthcare) and 0.45 μm (Millipore). The lysate was buffer exchanged into storage buffer (lysis buffer containing 0.02% (vol./vol.) Brij-35) over HiPrep 26/10 desalting columns to remove D-biotin from the lysate. Protein-containing fractions were pooled, supplemented with protease inhibitors and loaded onto a 1 mL HiTrap SP Sepharose FF column connected in tandem with 1 mL streptavidin mutein matrix beads (Sigma Aldrich) packed into a Tricorn 5/50 column (GE-Healthcare). The streptavidin mutein matrix column was washed with 30 mL storage buffer, 30 mL wash buffer (lysis buffer containing 200 mM KCl and 0.2% (vol./vol.) Brij-35) and 30 mL storage buffer. Proteins were eluted with storage buffer supplemented with 5 mM D-biotin. The buffer was then exchanged back into storage buffer using a HiTrap Desalting column. Protein-containing fractions were pooled and concentrated using Amicon centrifugal units (MWCO 30'000, Millipore), centrifuged (17,000xg, 10 min, 4°C) and separated by size exclusion chromatography using a Superose 6 10/300 GL column. γTuRC peak fractions were pooled, concentrated, ultracentrifuged (278,088.3xg, 10 min, 4°C), snap frozen and stored in liquid nitrogen. From 120 g of cell pellet typically ~85 μg of tagged γTuRC were purified.

Purification of Human chTOG-mGFP, mGFP-TPX2 and mGFP-EB3

GFP-tagged microtubule binders were purified as described (Roostalu et al., 2015, 2020). In brief, StrepTagII-chTOG-mGFP was expressed in Sf21 cells and affinity purified using a StrepTrap HP column. After removal of the N-terminal StrepTagII by tobacco etch virus (TEV) protease, chTOG-mGFP was further purified by size exclusion chromatography. StrepTagII-mGFP-TPX2 was expressed in Sf21 cells and affinity purified using a StrepTrap HP column. After removal of the N-terminal StrepTagII by TEV protease, mGFP-TPX2 was further purified by anion exchange chromatography and size exclusion chromatography. His_6-tagged mGFP-EB3 was expressed in E. coli (BL21 pRIL) and affinity purified using a HiTrap Chelating column. After removal of the N-terminal His_6-tag by TEV protease, mGFP-EB3 was further purified by size exclusion chromatography.

Tubulin Purification and Labelling

Porcine brain tubulin was purified and covalently labelled with NHS-biotin (Thermo Fisher) or NHS-CF640R (Sigma-Aldrich) using standard procedures (Castoldi and Popov, 2003; Hyman et al., 1991). CF640R-tubulin was labelled at a ratio of 0.4 fluorophores per tubulin dimer.

LC-MS/MS Analysis of Fluorescently Tagged γTuRC

Purified γTuRC was separated by SDS-PAGE and stained using InstantBlue (Expedeon). Protein bands were excised from the gel and analysed by the Francis Crick Institute Proteomics facility. Briefly, Tryptic peptides were analysed using a Q Exactive orbitrap mass spectrometer coupled to an Ultimate 3000 HPLC equipped with an EasySpray nano-source (Thermo Fisher Scientific). A one-hour method of MS1 orbitrap (60k resolution) followed by top 10 HCD MS2 (35k resolution) produced raw data files. Raw files were analysed in MaxQuant (v1.6.0.13) against the SwissProt Homo sapiens protein database (downloaded June 2019) using the iBAQ algorithm. The canonical GCP2 sequence was replaced with the construct sequence (GCP2-5xGly-TEV-5xGly-mBFP-5xGly-AviTag). Variable modifications of methionine oxidation and protein N-terminal acetylation along with a fixed modification of cysteine carbamidomethylation were selected. The proteingroups.txt file was imported in Perseus (v1.4.0.2) for data analysis. Potential contaminants, reverse sequences and proteins identified by site were removed. iBAQ intensities were log_2 transformed.

γTuRC-Mediated Microtubule Nucleation Assay

To study microtubule nucleation by γTuRC, we modified a previous TIRF microscopy-based surface nucleation assay without γTuRC (Roostalu et al., 2015). Flow chambers were assembled from one biotin-polyethylene glycol (PEG)-functionalized coverglass and one poly(L-lysine)-PEG-passivated counter glass.

Biotin-PEG-functionalized glass was prepared essentially as described (Bieling et al., 2010), with some modifications. In brief, 22 x 22 mm coverglasses (Menzel Gläser; #1.5) were sonicated in 3 M NaOH for 30 min, rinsed with Milli-Q water, sonicated in Piranha solution (95-97% H_2SO_4/30% H_2O_2 (3/2 (vol./vol.))) for 45 min in a fume hood, washed with Milli-Q water, sonicated for 5 min in Milli-Q water, and washed again in Milli-Q water. After spin-drying, sandwiches consisting of two coverglasses with (3-Glycidyloxypropyl) trimethoxy-silane (GOPTS) (Sigma Aldrich; 440167) in between them were kept at 75 °C for 30 min, left to cool for 15 min before glass sandwiches were separated. After being kept in acetone for 2 x 15 min, coverglasses were spin-dried and assembled into another sandwich with ~50 mg of PEG mix (biotin-CONH-PEG-NH_2 (Rapp Polymere; 133000-25-20)/HO-PEG-NH_2 (Rapp Polymere; 10300-20) (1/10 (w/w))), ensuring that the pre-functionalized sides of the glasses are on the inside of the sandwich. Sandwiches were kept at 75°C overnight after removing any air from the inside of the sandwich. After separation, coverglasses were sonicated for 30 min in Milli-Q water, washed with Milli-Q water, spin-dried and stored at 4°C for a maximum of 2 months.

Poly(L-lysine)-PEG-passivated counter glass was prepared by spreading 10 μL of 2 mg/mL Poly(L-lysine)-PEG (SuSoS) between two strips of double-sided tape (placed ~5 mm apart parallel to one another) on a microscopy glass (76x26 mm, VWR, 631-1550P) and left to dry for at least 20 min. The glass was washed with water and dried with N_2.

For a microscopy assay, a flow chamber consisting of one biotin-PEG-coverglass and a poly(L-lysine)-PEG counter glass was incubated for 10 min with 5% Pluronic F-127 (Sigma Aldrich) in MilliQ water, washed with assay buffer (AB: 80 mM PIPES, 60 mM KCl, 1 mM EGTA, 1 mM $MgCl_2$, 1 mM GTP, 5 mM 2-mercaptoethanol, 0.15% (w/vol.) methylcellulose (4,000 cP, Sigma-Aldrich) 1% (w/vol.) glucose, 0.02% (vol./vol.) Brij-35)) supplemented with 50 μg mL^{-1} κ-casein (Sigma-Aldrich), followed by a 3-min incubation with the same buffer additionally containing 50 μg mL^{-1} of NeutrAvidin (Life Technologies). The chamber was subsequently washed with γTuRC storage buffer and incubated for 5 min with prediluted γTuRC in γTuRC storage buffer to the concentration indicated for each experiment. Unbound γTuRC was removed by washing the flow cell with AB. Then the final assay mix was passed through, the chamber was sealed with vacuum grease (Beckman) and placed onto the microscope.

Final assay mix: AB supplemented with oxygen scavengers (160 μg mL^{-1} catalase (Sigma-Aldrich), 680 μg mL^{-1} glucose oxidase (Serva)) diluted in BRB80 (80mM PIPES, 1mM EGTA, 1mM $MgCl_2$), 1 mg ml^{-1} bovine serum albumin (Sigma-Aldrich) in BRB80, varying concentrations of tubulin (containing 4.8% CF640R-labelled tubulin). For experiments with microtubule binders 2.9% (vol./vol.) of either chTOG-mGFP, mGFP-TPX2 or mGFP-EB3 was added at different concentrations. chTOG-mGFP and mGFP-TPX2 concentrations were altered by predilution in their storage buffers (Jha et al., 2017; Roostalu et al., 2015). mGFP-EB3 was diluted in BRB80. The final assay mix containing chTOG-GFP was ultracentrifuged (278,088.03xg, 10 min, 4°C) before flowing the mix into the chamber. To keep the buffer composition of the final assay mix unchanged within a set of experiments and to allow for direct comparisons between experiments, the overall BRB80 and storage buffer content was kept constant within one set of experiments.

Microtubule Dynamics Assays Using 'Seeds'

To image the properties of microtubules having both dynamic plus and minus ends, microtubules were grown from pre-polymerized and immobilized GMPCPP-stabilized microtubules ('seeds'). Dynamics assays were performed as nucleation assays, but instead of γTuRC biotinylated and fluorescently labelled microtubule seeds were bound to the glass surface. Seeds were prepared as described previously (Bieling et al., 2010), here containing 39% CF640R-labelled tubulin.

In brief, 6.7 μM tubulin, 5 μM biotinylated tubulin and 7.1 μM CF640R-labelled tubulin and 0.5 μM GMPCPP (Jena bioscience, NU-405S) in BRB80 was incubated for 1 h at 37°C, diluted 8.33-fold with prewarmed BRB80 and centrifuged at 17,000 g at room temperature for 10 min. The pellet was resuspended in prewarmed BRB80 and centrifuged at 17,000 g for 2 min, followed again by resuspension of the pellet in prewarmed BRB80. Microtubule seeds were kept at room temperature and used on the same day.

TIRF Microscopy

All experiments were performed using a total internal reflection fluorescence (TIRF) microscope (Cairn Research, Faversham, UK) (Hannabuss et al., 2019). Experiments were imaged 2 min after placing the chamber on the microscope. The temperature was kept at 33±1°C for all experiments. Two- and three-colour time-lapse imaging for γTuRC nucleation assays and dynamics assays were performed at 1 frame/5 s with a 300-ms exposure time for tubulin (640 nm) and GFP (480 nm) channels and 1000-ms for γTuRC-mBFP (408 nm) using a 60x 1.49 NA Nikon objective lens. For single molecule γTuRC assays shown in Figure 2F, images were acquired at 1 frame/1.8 s with a 500-ms exposure time using a 100x 1.49 N.A. Nikon objective lens. CF640R-tubulin (640 nm excitation) and mGFP-tagged proteins (488 nm excitation) were imaged simultaneously. γTuRC-mTagBFP-BAP (405 nm excitation) was imaged every 10 frames for single molecule γTuRC assays and once at the beginning and at the end of the movie for γTuRC nucleation assays.

TIRF Microscopy Image Processing

The Fiji package of ImageJ was used to generate kymographs (space-time plots) and to merge image sequences from different channels. For multi-colour imaging, image alignment was performed using a Matlab script (Maurer et al., 2014). Background was subtracted using the background subtraction tool of Fiji ('rolling ball' method). For movies from single molecule γTuRC assays shown in Figure 2F γTuRC-mTagBFP-AviTag images were merged using the 'grouped Z project' function in Fiji. To subtract camera noise an empty flow chamber was imaged using the same imaging conditions. The background image was generated as described above and subtracted from the γTuRC-mTagBFP-AviTag image, which was then used to merge with images of CF640R-tubulin.

Microtubule Growth Speeds

Growth speeds were measured directly from kymographs using the 'Resclice function' in Fiji. Lines were drawn manually along growing plus- and minus-ends. Growth speeds were calculated from the slope of the line. The total number of microtubules used for the measurement of growth speeds for each experimental condition is stated in the corresponding figure legend and data was pooled from at least three independent experiments if not stated otherwise. For conditions with high nucleation rates, at least 50 microtubules per experimental repeat were analysed. For condition with low nucleation rates, all microtubules with a minimum lifetime of ~2 min were used for analysis.

Microtubule Nucleation Rate Analysis

For each nucleation assay, microtubules were counted manually at 10 different time points either until the end of the movie or until individual microtubule nucleation events could no longer be identified due to overcrowding. The total number of nucleated microtubules in a field of view at a given time point was obtained by counting the newly nucleated microtubules and adding it to the number obtained at the previous analysed time point. Microtubule numbers were tracked using the 'Point tool' together with the 'ROI manager tool' in Fiji. For the quantification of γTuRC-mediated microtubule nucleation rates, only microtubules were counted that started nucleating from the surface and that stayed surface-attached. Microtubule nucleation rates represent the slope of the linear regression for each condition and are given in number per nucleated microtubules per field of view and per time.

Negative Stain Grid Preparation and Data Collection

A 4-μl droplet of human γTuRC (purified as described above) diluted in γTuRC storage buffer was applied to a freshly glow-discharged carbon-coated grid (C300Cu100, EM Resolution) and incubated for 2 min. The grid was stained with consecutive applications onto three 50-μl droplets of 2% uranyl acetate solution for 30 s each. The grid was then blotted dry and stored until imaged on a 120 keV G2 Spirit transmission electron microscope (FEI) equipped with a 2k×2k Ultrascan-1000 camera (Gatan). The Micrographs were collected using a nominal magnification of 30,000x, resulting in a pixel size of 3.45 Å at the specimen level.

Cryo Grid Preparation and Data Collection

Freeze-thawed human γTuRC (purified as described above) was briefly spun to remove aggregates. Lacey grids (400 mesh) with a layer of ultra-thin carbon (Agar Scientific) were glow-discharged at 45 mA for 1 min using a K100X Glow Discharge Unit (EMS). A 4 μl-droplet was then applied directly onto the carbon-side of the grid loaded into the humidity chamber of a Vitrobot Mark IV (Thermo Fisher) set to room temperature and 90% humidity. After an incubation time of 60 seconds, the grid was blotted for 3s and plunged into liquid ethane. The ice quality was assessed on a 200 kV Talos Arctica (Thermo Fisher) and a small dataset was collected to evaluate the sample quality.

The highest-quality grid was imaged using a 300kV Titan Krios electron microscope (Thermo Fisher) using a GIF Quantum energy filter (Gatan) and a K2 Summit direct detector (Gatan), operated in counting mode. A total of 2,4000 movies were collected over two sessions at a pixel size of 1.08 Å/px with a total dose of ~50 e−/A2 and a defocus range of -1.0 - -3.5 μm.

Negative Stain Electron Microscopy Image Processing

The particles were picked using e2boxer.py of the EMAN2 v2.07 software package (Tang et al., 2007), using the semi-automated (swarm) option. Box files were then imported in the RELION-3.0 (Zivanov et al., 2018), which was used for all downstream image processing steps that were performed. Contrast transfer function parameters were determined using Gctf v.1.18 (Zhang, 2016), and extracted particles were subjected to two-dimensional classification.

Cryo-EM Image Processing

To correct for beam-induced movements all movie frames were aligned using dose-weighted averaging in MotionCor2(Zheng et al., 2017). CTF parameters were estimated using non-dose-weighted micrographs generated by Gctf v.1.18 (Zhang, 2016). Automated particle-picking was performed using crYOLO of the SPHIRE software package (Wagner et al., 2019). Box files were imported in RELION-3 (Zivanov et al., 2018) and a total of ~ 1.1 million particles were initially binned by a factor of four and extracted from dose-weighted micrographs with a box size of 128 pixels. After several rounds of two-dimensional classification, a total of 522,496 high-resolution particles were selected, which evidently contained high-resolution information. Unbinned particles were re-extracted, using a 512-pixel box size. These particles were used to generate three reference free *ab initio* models using cryoSPARC v2 (Punjani et al., 2017). The best model, which resulted from 229,744 particles, was imported in RELION-3, filtered to 60 Å and used as a starting reference for 3D classification of the 522,496 high-resolution particles. The combination of all particles yielded in the highest resolution class, which was subsequently subjected to one initial 3D refinement, followed by three rounds of CTF refinement and one Bayesian particle polishing step. Polished particles were subjected to one final round of CTF refinement, 3D refinement and post processing, yielding in a final 3D structure with an overall resolution of 4 Å. Further cryoEM density modification implemented in Phenix (Terwilliger et al., 2019) increased the resolution to 3.7 Å (used for display in figures showing amino acid chains). Although BFP was present in the complex, fused to the C-terminus of GCP2, density for this tag was not visible in the cryo-EM structure, due to both flexibility and the mixture of tagged and untagged GCP2 found in the complex.

Generation of an Atomic Model

The crystal structure of human GCP4 (PDB entry 3RIP) (Guillet et al., 2011) was separated in three distinct domains and used for docking into the cryo-EM map, using the Fit in map option in UCSF Chimera (Pettersen et al., 2004). Highest correlation GCP subunits were assigned to GCP4, while GCP3 was recognised because of a characteristic helical extension in the C-terminal γ-tubulin interacting domain, first modelled using iTasser (Roy et al., 2010), adjusted manually in Coot (Emsley et al., 2010) and refined using Phenix (Afonine et al., 2018) and Namdinator (Kidmose et al., 2019). Other GCP assignments were based on CLMS results (detailed below).

Crosslinking and Mass Spectrometry

The purified γTuRC complex at a concentration of 0.2 mg/ml in gel filtration buffer (50 mM HEPES pH 7.4, 150 mM KCl, 1 mM $MgCl_2$, 1 mM EGTA, 1 mM DTT, 0.1 mM GTP and 0.02 % Brij-35) was crosslinked with 2.4 mM disulfosuccinimidyl suberate (BS3) in a thermomixer for 1 h at 24°C and 850 rpm. The reaction was quenched with 92 mM NH_4HCO_3 in a thermomixer for 30 min at 24°C and 850 rpm. The crosslinked sample was cold-acetone precipitated. The dried protein pellet was resolubilized in 40 μL digestion buffer (8M urea in 100 mM ammonium bicarbonate (ABC) with 1 mM Dithiothreitol (DTT)) to an estimated protein concentration of 1 mg/mL. Dissolved protein sample was reduced by addition of 0.2 uL 1M DTT, the reduction reaction was incubated at room temperature for 30 minutes. The free -SH groups in the sample were then alkylated by adding 1.2 uL 500 mM Iodoacetamide (IAA) and incubating at room temperature for 20 minutes. After alkylation, 0.2 uL 1M DTT was added to quench excess of IAA. Subsequently, protein sample was digested with LysC (with 1:50 (m/m) protein to protease ratio) at room temperature for four hours. The sample was then diluted with 100 mM ABC to reach urea concentration of 1.5 M. Trypsin was added with 1:50 (m/m) protein to protease ratio to further digest proteins for over night (~15 hours) at room temperature. Resulting peptides were de-salted using C18 StageTips (PMID:17703201).

20% of total peptides were directly analysed by liquid chromatography–tandem mass spectrometry (LC-MS/MS) in duplicate. The remaining 80% peptides were fractionated using size exclusion chromatography in order to enrich for crosslinked peptides (PMID:24356771). Peptides were separated using a Superdex Peptide 3.2/300 column (GE Healthcare) at a flow rate of 10 μl/min. The mobile phase consisted of 30% (v/v) acetonitrile and 0.1% trifluoroacetic acid. The earliest six peptide-containing fractions (50 μl each) were collected. Solvent was removed using a vacuum concentrator. The fractions were then analysed by LC-MS/MS.

LC-MS/MS analysis was performed using an Orbitrap Fusion Lumos Tribrid mass spectrometer (Thermo Fisher Scientific), connected to an Ultimate 3000 RSLCnano system (Dionex, Thermo Fisher Scientific). Each SEC fraction was resuspended in 1.6% v/v acetonitrile 0.1% v/v formic acid and analysed with two LC-MS/MS acquisitions. Peptides were injected onto a 50-centimetre EASY-Spray C18 LC column (Thermo Scientific) that is operated at 50°C column temperature. Mobile phase A consists of water, 0.1% v/v formic acid and mobile phase B consists of 80% v/v acetonitrile and 0.1% v/v formic acid. Peptides were loaded and separated at a flowrate of 0.3 μL/min. Eluted peptides were ionized by an EASY-Spray source (Thermo Scientific) and introduced directly into the mass spectrometer.

For non-fractionated samples, peptides were separated using a linear gradient going from 2% mobile phase B to 40% mobile phase B over 110 minutes, followed by a linear increase from 40% to 95% mobile phase B in eleven minutes. The MS data is acquired in the data-dependent mode with three-second acquisition cycle. The full scan mass spectrum was recorded in the Orbitrap with a resolution of 120,000. The ions with a charge state from 3+ to 7+ were isolated and fragmented using higher-energy collisional dissociation (HCD) with 30% collision energy. The fragmentation spectra were then recorded in the Orbitrap with a resolution of 50000. Dynamic exclusion was enabled with single repeat count and 60-second exclusion duration.

The collected SEC fractions were each analysed with duplicated acquisitions. Peptides were separated by applying a gradient ranging from 2% to 45% B over 90 min. Gradient was optimized for each corresponding SEC fraction. Following the separating gradient, the content of B was ramped to 55% and 95% within 2.5 minutes each. The MS data is acquired in the data-dependent mode with the top-speed option. For each three-second acquisition cycle, the full scan mass spectrum was recorded in the Orbitrap with a resolution of 120,000. The ions with a charge state from 3+ to 7+ were isolated and fragmented using Higher-energy collisional dissociation (HCD). For each isolated precursor, one of three collision energy settings (26%, 28% or 30%) was selected for fragmentation using data dependent decision tree based on the m/z and charge of the precursor. The fragmentation spectra were then recorded in the Orbitrap with a resolution of 50000. Dynamic exclusion was enabled with single repeat count and 60-second exclusion duration.

The MS2 peak lists were generated from the raw mass spectrometric data files using the MSConvert module in ProteoWizard (version 3.0.11729). The default parameters were applied, except that Top MS/MS Peaks per 100 Da was set to 20 and the de-noising function was enabled. Precursor and fragment m/z values were recalibrated. Identification of crosslinked peptides was carried out using xiSEARCH software (https://www.rappsilberlab.org/software/xisearch) (PMID:31556486). Peak lists from all LC-MS/MS acquisitions were searched against the sequence and the reversed sequence of γTuRC subunits. The following parameters were applied for the search: MS accuracy = 5 ppm; MS2 accuracy = 10 ppm; enzyme = trypsin (with full tryptic specificity); allowed number of missed cleavages = two; missing monoisotopic peak=2 5; cross-linker = BS3 the reaction specificity for BS3 was assumed to be for lysine, serine, threonine, tyrosine and protein N termini); fixed modifications = carbamidomethylation on cysteine; variable modifications = oxidation on methionine, modifications by BS3 that are hydrolyzed or amidated on the end. Identified crosslinked peptide candidates were filtered using XiFDR (PMID: 28267312). A false discovery rate (FDR) of 2% on residue-pair-level was applied with "boost between" option selected. A list of identified crosslinked residue pairs is reported in Data S2. Structural interpretation of interprotein crosslinks was focused on protein pairs that were crosslinked with three or more residue pairs. The pseudo-atomic model of γTuRC complex was compared against the crosslinking data. The distances between the Cα atoms of crosslinked residue pairs in the

CellPress
OPEN ACCESS

model were measured and compared against a theoretical crosslinking limit of 30 Å for crosslinker BS3 (calculated based the spacer of the crosslinker and the length of the side chains of crosslinked residues).

QUANTIFICATION AND STATISTICAL ANALYSIS

Image analysis was performed with the Fiji package of ImageJ and Matlab. Plots were generated in GraphPad Prism. Data were pooled from at least three independently performed experiments if not stated otherwise. All error bars represent the standard error of mean (s.e.m.) or standard deviation (s.d.) as indicated in each Figure. Linear regression and curve fitting were performed using GraphPad Prism. Details of the analysis are given in the STAR Methods DETAIL section.

Developmental Cell, Volume *53*

Supplemental Information

Microtubule Nucleation Properties of Single Human γTuRCs Explained by Their Cryo-EM Structure

Tanja Consolati, Julia Locke, Johanna Roostalu, Zhuo Angel Chen, Julian Gannon, Jayant Asthana, Wei Ming Lim, Fabrizio Martino, Milos A. Cvetkovic, Juri Rappsilber, Alessandro Costa, and Thomas Surrey

Figure S1

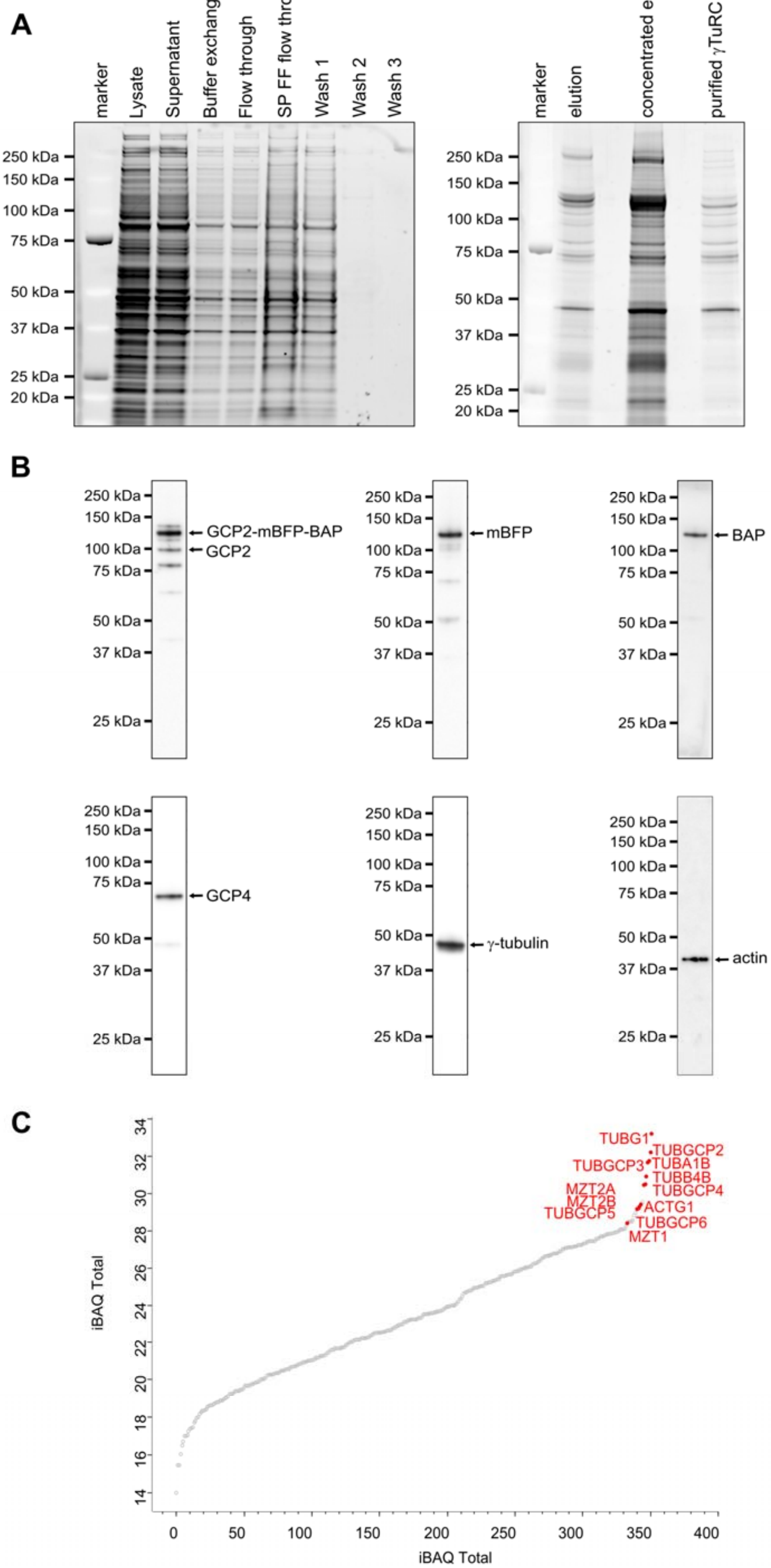
A
marker
Lysate
Supernatant
Buffer exchange
Flow through
SP FF flow through
Wash 1
Wash 2
Wash 3
250 kDa
150 kDa
100 kDa
75 kDa
50 kDa
37 kDa
25 kDa
20 kDa
marker
elution
concentrated elution
purified γTuRC
B
GCP2-mBFP-BAP
GCP2
mBFP
BAP
GCP4
γ-tubulin
actin
C
TUBG1
TUBGCP2
TUBGCP3
TUBA1B
TUBB4B
TUBGCP4
MZT2A
MZT2B
TUBGCP5
ACTG1
TUBGCP6
MZT1
iBAQ Total
iBAQ Total

Figure S1. Purification of tagged human γTuRC (Related to Fig. 1). (A) Sypro-Ruby stained SDS-PAGE showing the purification of γTuRC-mBFP-BAP purification from human cells. **(B)** Uncropped western blots of purified γTuRC using antibodies against GCP2, mBFP, GCP4, γ-tubulin and actin. Biotinylation of the biotin acceptor peptide (BAP) was assessed by immunoblotting using horse radish peroxidase (HRP)-coupled streptavidin. **(C)** Scatter plot representing the protein density by iBAQ intensity. Rank vs log10 ibaq value ordered from lowest to highest estimated relative abundance. TUBG1 was the most abundant protein in the prep.

Figure S2

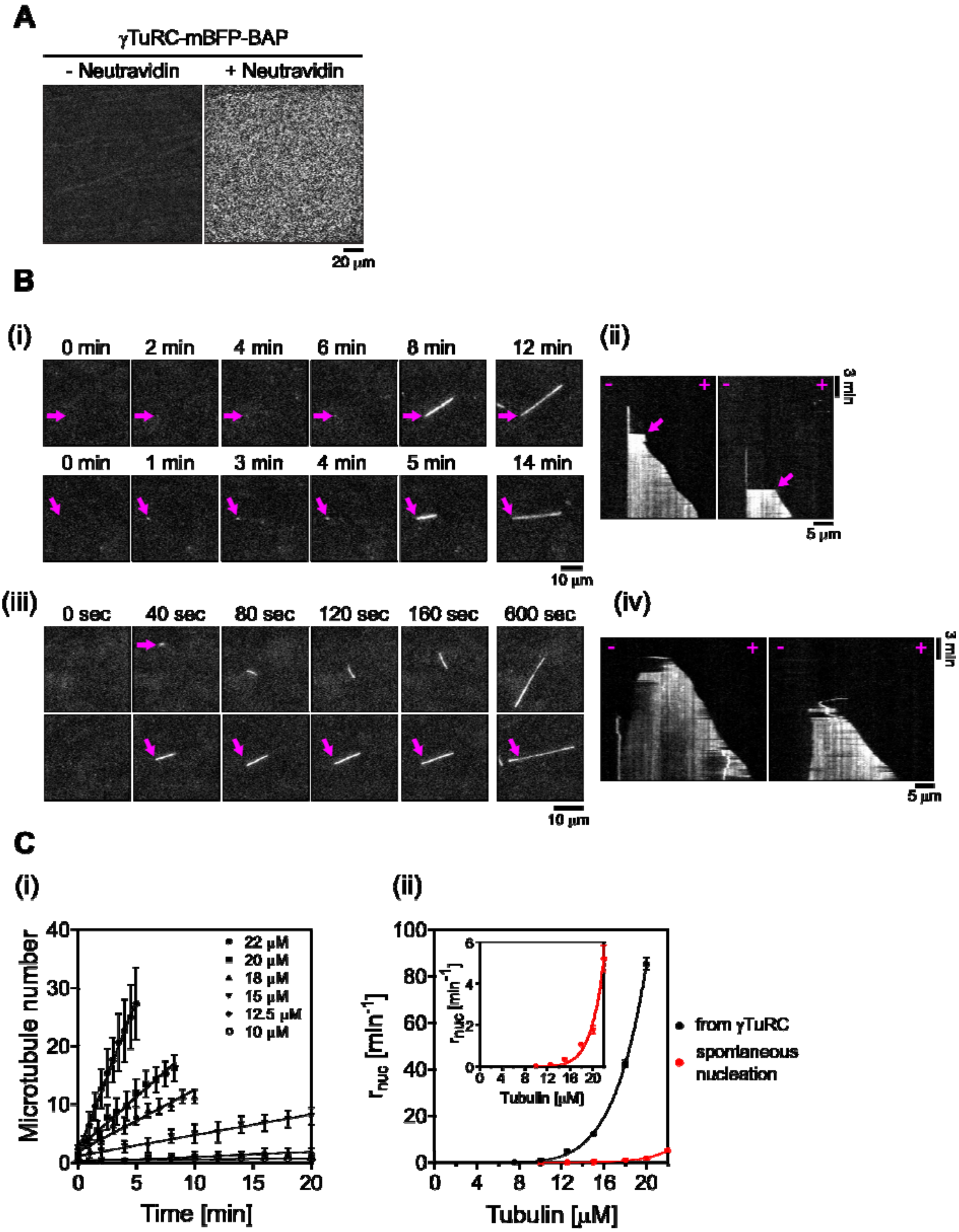
A
γTuRC-mBFP-BAP
- Neutravidin
+ Neutravidin
20 µm
B
(i)
0 min
2 min
4 min
6 min
8 min
12 min
0 min
1 min
3 min
4 min
5 min
14 min
10 µm
(ii)
3 min
5 µm
(iii)
0 sec
40 sec
80 sec
120 sec
160 sec
600 sec
10 µm
(iv)
3 min
5 µm
C
(i)
Microtubule number
Time [min]
22 µM
20 µM
18 µM
15 µM
12.5 µM
10 µM
(ii)
r_nuc [min^-1]
Tubulin [µM]
from γTuRC
spontaneous nucleation

Figure S2. Characterization of TIRF-microscopy based microtubule nucleation assay in the presence and absence of γTuRC (Related to Figs. 1, 2 and 3). (A) Specificity of γTuRC immobilization on functionalized glass surfaces. Biotinylated fluorescent γTuRC (373 pM used for immobilization) was bound to functionalized glass surfaces pre-incubated with NeutrAvidin or in the absence of NeutrAvidin as indicated. Representative TIRFM images of γTuRC-mBFP fluorescence are shown, indicating that γTuRC surface immobilization is specific. No tubulin was added. Field of view was always 164 µm x 164 µm. Fluorescence intensities are directly comparable. Scale bars as indicated. **(B)** Examples of 'falling' γTuRC-nucleated microtubules and 'landing' spontaneously nucleated microtubules in solution. **(i)** Representative time series of two γTuRC-nucleated microtubules that first grow out of the TIRF field and then 'fall' onto the surface (15 µM CF640R-tubulin, 93 pM γTuRC, 33°C). 'Falling' microtubules only grow from one end while the other end is stably anchored to the surface indicating that these microtubules are initiated by surface immobilized γTuRC. Purple arrows indicate the stably anchored microtubule ends. **(ii)** Corresponding TIRFM kymographs. Purple arrow marks 'falling' event. **(iii)** Representative time series of two spontaneously nucleated microtubules, 'landing' from solution (15 µM CF640R-tubulin, 47 pM γTuRC, 33°C). Spontaneously nucleated microtubules are distinguishable from γTuRC-nucleated microtubules because they are not anchored to the surface (top row, purple arrow mark first appearance of spontaneously nucleated microtubule) and grow with both ends (bottom row, purple arrows mark slow growing minus-end). **(iv)** Corresponding TIRFM kymographs. Scale bars as indicated. t=0 is 2 min after placing the sample at 33°C. **(C)** Dependence of spontaneous microtubule nucleation in the absence of γTuRC on tubulin concentration. **(i)** Microtubule nucleation at 33°C in the absence of γTuRC at varying CF640R-tubulin concentrations (10 µM, 12.5 µM, 15 µM, 18 µM, 20 µM, 22 µM). Plot is showing the linear increase in microtubule number over time. No microtubules were nucleated below 10 µM tubulin. Lines represent a linear regression. **(ii)** Plot showing the mean microtubule nucleation rate at different tubulin concentrations in the presence of γTuRC (see Fig. 3d (iv), black) and the mean rate of spontaneous microtubule nucleation in the absence of γTuRC at different tubulin concentrations (red, and inset). Data for plots were pooled from at least three independent experiments. The plots were fit using a power law function. Nucleation rates (r_{nuc}) were taken from the slope of the linear regression of the increase of microtubule number over time (see Fig. S6a and Fig. 3d (i)). All error bars are s.e.m. For symbols without visible error bars, error bars are smaller than the symbol size. t=0 is 2 min after placing the sample at 33 °C.

Figure S3

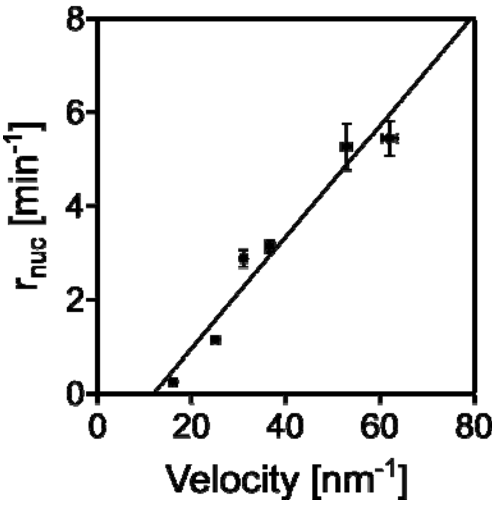

Figure S3. Linear relationship between γTuRC-mediated microtubule nucleation and chTOG-accelerated microtubule plus-end growth (Related to Fig. 4). Plot of the mean nucleation rate (r_{nuc}) against the mean microtubule plus-end growth speed at different chTOG-mGFP concentrations (6 nM, 13 nM, 25 nM, 50 nM, 100 nM). Assays were performed as described in Fig.4 a-d. Nucleation rates (r_{nuc}) were taken from Fig. 4b and microtubule growth speeds from Fig. 4d. Line represents the linear regression. Error bars are s.e.m. For symbols without visible error bars, error bars are smaller than the symbol size.

Figure S4

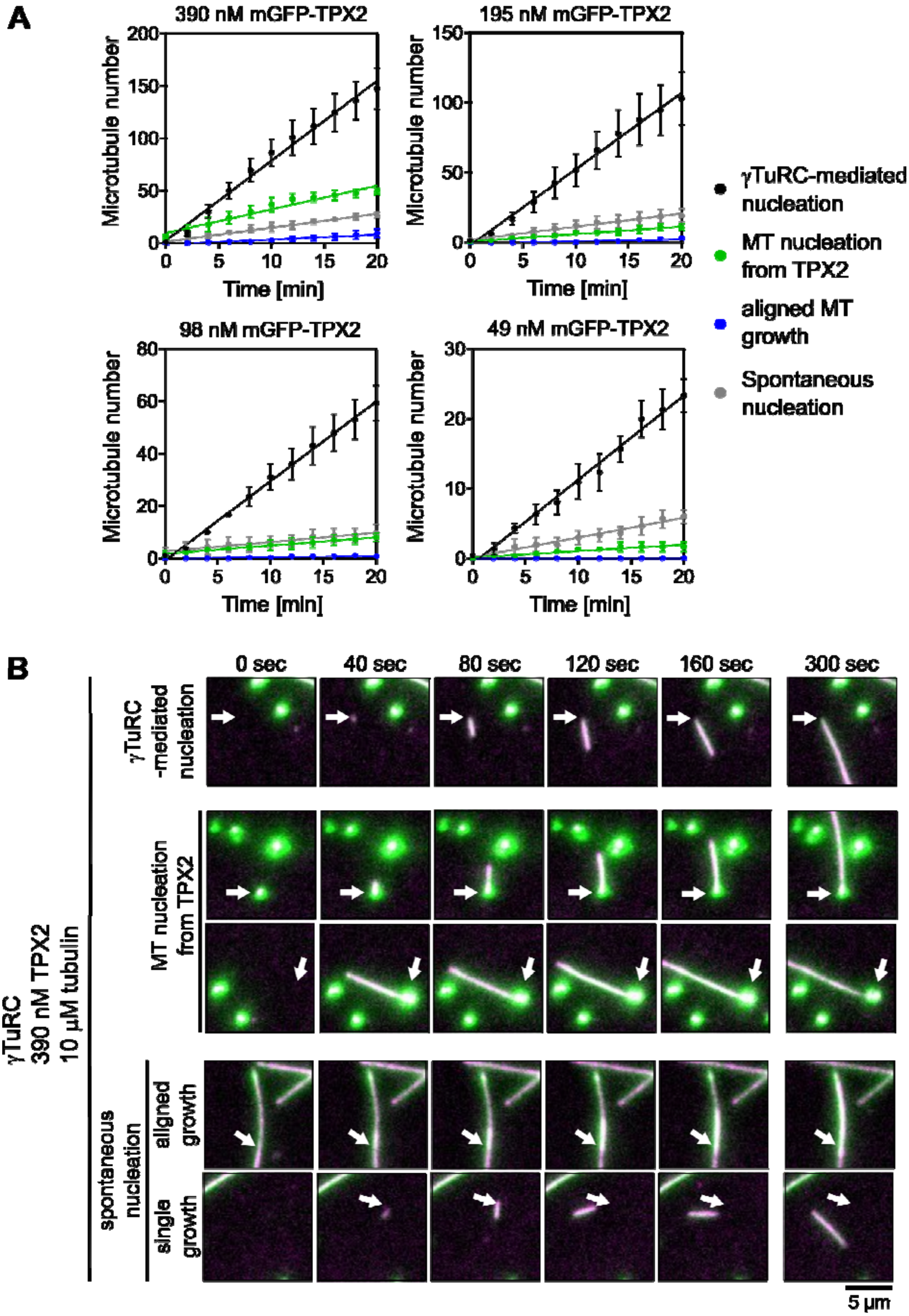
A
390 nM mGFP-TPX2
195 nM mGFP-TPX2
98 nM mGFP-TPX2
49 nM mGFP-TPX2
Microtubule number
Time [min]
γTuRC-mediated nucleation
MT nucleation from TPX2
aligned MT growth
Spontaneous nucleation
B
0 sec
40 sec
80 sec
120 sec
160 sec
300 sec
γTuRC -mediated nucleation
MT nucleation from TPX2
spontaneous nucleation
aligned growth
single growth
γTuRC
390 nM TPX2
10 μM tubulin
5 μm

Figure S4. Microtubules nucleated in assays performed in the presence of mGFP-TPX2 (Related to Fig. 4). (A) Plots showing linearly increasing nucleated microtubule numbers over time at different mGFP-TPX2 concentrations (49 nM, 98 nM, 195 nM, 390 nM). Assays were performed at 33°C in the presence of the indicated mGFP-TPX2 concentration and 10 µM tubulin. 373 pM γTuRC were used for immobilization. Representative TIRFM images of microtubules (magenta) nucleated in presence of γTuRC and 390 nM TPX2 (green) are shown in **(B)**. Nucleated microtubules were classified into 4 categories: (1) γTuRC-mediated nucleation (black line, first row of images), (2) microtubules nucleating from TPX2 (green line) are microtubules growing from TPX2 accumulations on the surface (second row of images) or TPX2 accumulations which land on the surface after forming a microtubule (third row of images), (3) spontaneously nucleated microtubules which grow aligned with an existing microtubule (fourth row of images) and (4) and spontaneously nucleated microtubules which elongate and diffuse on the surface (fifth row of images). Lines represent the linear regression. All error bars are s.e.m. For symbols without visible error bars, error bars are smaller than the symbol size. Field of view was always 164 µm x 164 µm. Fluorescence intensities are directly comparable. Scale bars as indicated. t=0 is 2 min after placing the sample at 33 °C.

Figure S5

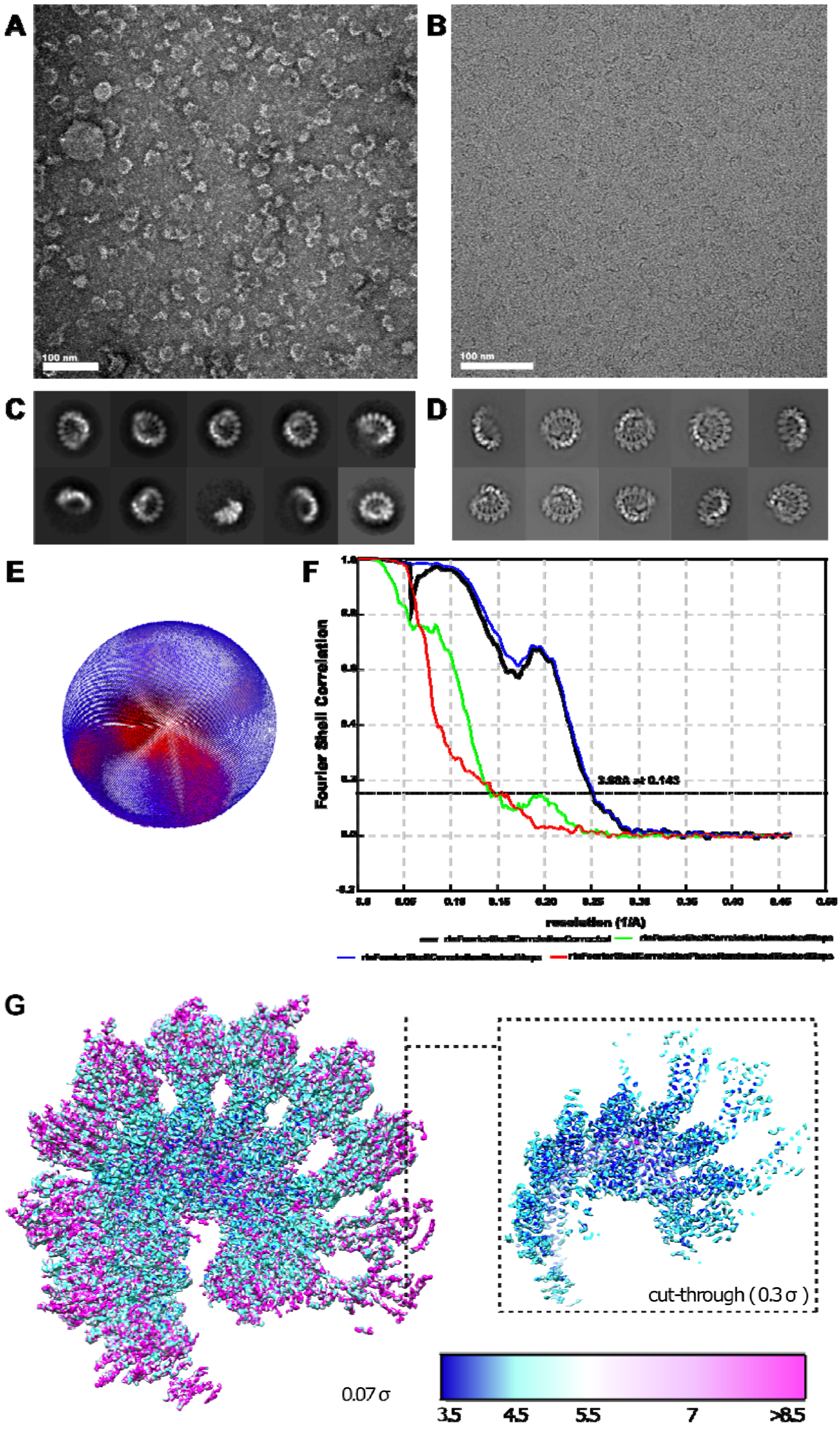
A
B
100 nm
100 nm
C
D
E
F
Fourier Shell Correlation
resolution (1/A)
G
cut-through (0.3 σ)
0.07 σ
3.5
4.5
5.5
7
>8.5

Figure S5. Electron microscopy image acquisition and processing (Related to Figs. 5 and 6). (A) Micrograph of a negatively stained sample. **(B)** Cryo-electron micrograph of a frozen-hydrated sample. **(C)** 2D class averages deriving from negative stain images. **(D)** 2D class averages deriving from cryo-EM images. **(E)** Angular distribution of particles used in the final cryo-EM reconstruction. **(F)** Fourier shell correlation indicating a resolution of 4 Å according to the 0.143 criterion. **(G)** Cryo-EM map colored according to the local resolution (after RELION3 postprocessing), as determined by using ResMap.

Figure S6

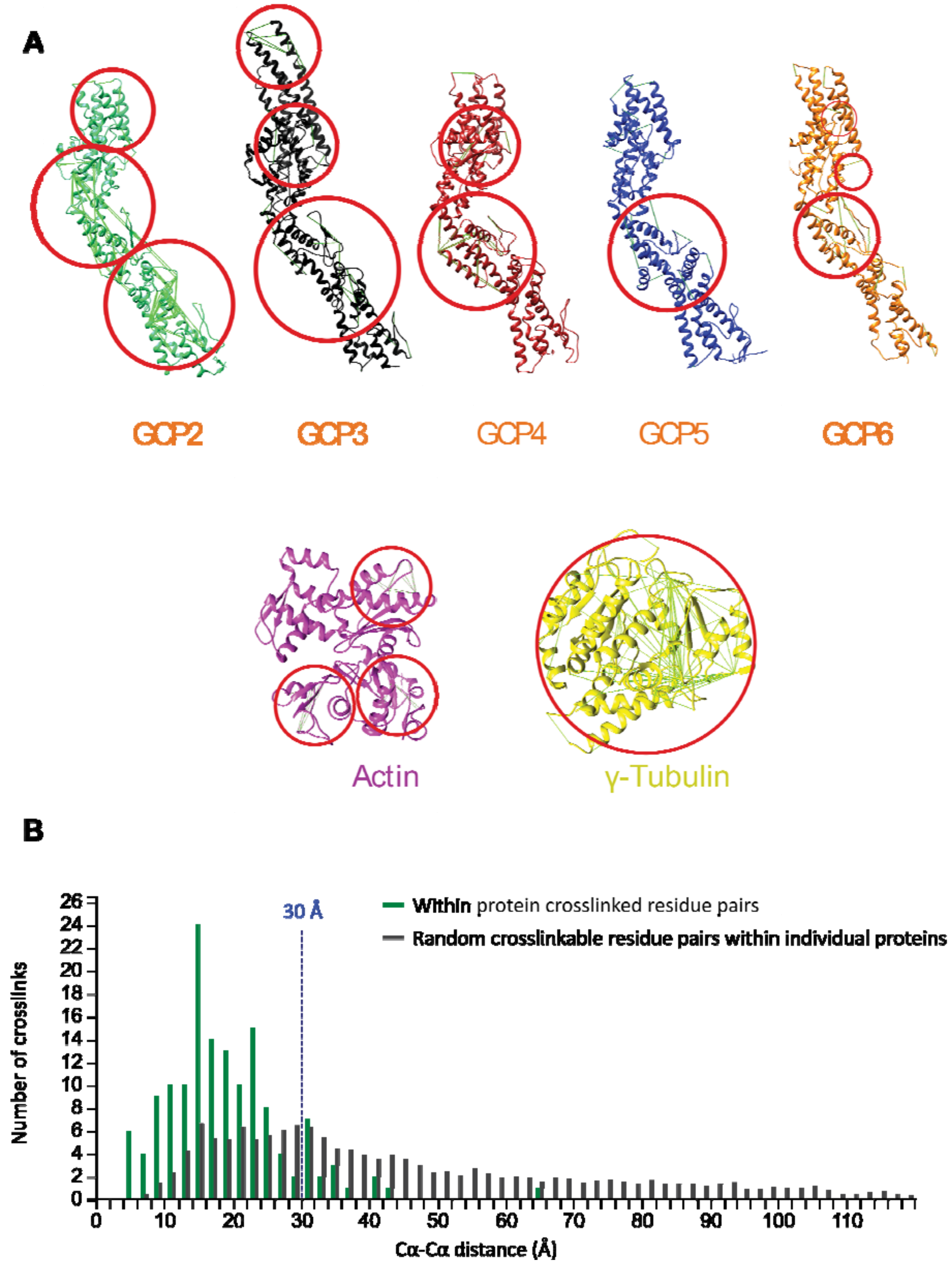

Figure S6. Summary of CLMS results (Related to Figs. 6 and 7). (A) Intramolecular crosslinks detected in CLMS experiments. Crosslinks are highlighted with a green silhouette. Red circles indicate clusters of crosslinks that measure 3 nm or shorter (compatible with the linker length of the BS3 crosslinker). **(B)** Distance distribution of within protein crosslinks detected in γTuRC complex (n=146). Theoretical crosslinking limit (30 Å) is indicated.
